# Comparison of Six Lytic Polysaccharide Monooxygenases from *Thermothielavioides terrestris* Shows That Functional Variation Underlies the Multiplicity of LPMO Genes in Filamentous Fungi

**DOI:** 10.1128/aem.00096-22

**Published:** 2022-03-22

**Authors:** Monika Tõlgo, Olav A. Hegnar, Heidi Østby, Anikó Várnai, Francisco Vilaplana, Vincent G. H. Eijsink, Lisbeth Olsson

**Affiliations:** a Division of Industrial Biotechnology, Chalmers University of Technology, Gothenburg, Sweden; b Wallenberg Wood Science Centre, Department of Biology and Biological Engineering, Chalmers University of Technology, Gothenburg, Sweden; c Faculty of Chemistry, Biotechnology and Food Science, Norwegian University of Life Sciences, Ås, Norway; d Division of Glycoscience, Department of Chemistry, KTH Royal Institute of Technology, Stockholm, Sweden; e Wallenberg Wood Science Centre, KTH Royal Institute of Technology, Stockholm, Sweden; Nanjing Agricultural University

**Keywords:** *Thermothielavioides terrestris*, *Thielavia terrestris*, filamentous fungi, biomass degradation, LPMO, AA9, hemicellulose, cellulose, plant cell wall, xylan, lytic polysaccharide monooxygenases

## Abstract

Lytic polysaccharide monooxygenases (LPMOs) are mono-copper enzymes that oxidatively degrade various polysaccharides. Genes encoding LPMOs in the AA9 family are abundant in filamentous fungi while their multiplicity remains elusive. We describe a detailed functional characterization of six AA9 LPMOs from the ascomycetous fungus *Thermothielavioides terrestris* LPH172 (syn. *Thielavia terrestris*). These six LPMOs were shown to be upregulated during growth on different lignocellulosic substrates in our previous study. Here, we produced them heterologously in Pichia pastoris and tested their activity on various model and native plant cell wall substrates. All six *T. terrestris* AA9 (*Tt*AA9) LPMOs produced hydrogen peroxide in the absence of polysaccharide substrate and displayed peroxidase-like activity on a model substrate, yet only five of them were active on selected cellulosic substrates. *Tt*LPMO9A and *Tt*LPMO9E were also active on birch acetylated glucuronoxylan, but only when the xylan was combined with phosphoric acid-swollen cellulose (PASC). Another of the six AA9s, *Tt*LPMO9G, was active on spruce arabinoglucuronoxylan mixed with PASC. *Tt*LPMO9A, *Tt*LPMO9E, *Tt*LPMO9G, and *Tt*LPMO9T could degrade tamarind xyloglucan and, with the exception of TtLPMO9T, beechwood xylan when combined with PASC. Interestingly, none of the tested enzymes were active on wheat arabinoxylan, konjac glucomannan, acetylated spruce galactoglucomannan, or cellopentaose. Overall, these functional analyses support the hypothesis that the multiplicity of the fungal LPMO genes assessed in this study relates to the complex and recalcitrant structure of lignocellulosic biomass. Our study also highlights the importance of using native substrates in functional characterization of LPMOs, as we were able to demonstrate distinct, previously unreported xylan-degrading activities of AA9 LPMOs using such substrates.

**IMPORTANCE** The discovery of LPMOs in 2010 has revolutionized the industrial biotechnology field, mainly by increasing the efficiency of cellulolytic enzyme cocktails. Nonetheless, the biological purpose of the multiplicity of LPMO-encoding genes in filamentous fungi has remained an open question. Here, we address this point by showing that six AA9 LPMOs from a single fungal strain have various substrate preferences and activities on tested cellulosic and hemicellulosic substrates, including several native xylan substrates. Importantly, several of these activities could only be detected when using copolymeric substrates that likely resemble plant cell walls more than single fractionated polysaccharides do. Our results suggest that LPMOs have evolved to contribute to the degradation of different complex structures in plant cell walls where different biomass polymers are closely associated. This knowledge together with the elucidated novel xylanolytic activities could aid in further optimization of enzymatic cocktails for efficient degradation of lignocellulosic substrates and more.

## INTRODUCTION

Lignocellulose is the most abundant polymeric composite on Earth and is a recalcitrant but promising renewable substrate for industrial biotechnology applications (1). In nature, the decomposition of lignocellulosic biomass plays a key role in the global carbon cycle and is primarily performed by fungi (2). The depolymerization of the complex plant cell wall, predominantly composed of cellulose, lignin, and various hemicelluloses, requires a large suite of enzymes that work in concert on the different components. In fungi, the plant cell wall polysaccharides are primarily degraded by secreted glycoside hydrolases (GHs), carbohydrate esterases, polysaccharide lyases, and lytic polysaccharide monooxygenases (LPMOs) (3–6). LPMOs are monocopper enzymes that depolymerize crystalline and amorphous polysaccharides via the oxidation of scissile α- or β-(1→4)-glycosidic bonds (7–13). The copper in the active site is coordinated by two highly conserved histidines commonly referred to as the histidine brace (8, 9, 14). Catalysis by LPMOs requires the reduction of the active-site copper from Cu(II) to Cu(I) by a reducing agent and H_2_O_2_ (or O_2_) as a cosubstrate (7, 15–17). The introduction of a hydroxyl group at either the C_1_ or C_4_ position by the LPMO leads to spontaneous bond cleavage and formation of a lactone (C_1_ oxidation) or a 4-ketoaldose (C_4_ oxidation), which are in a pH-dependent equilibrium with their hydrated forms, being an aldonic acid or a gemidiol, respectively (9, 18). In addition to cleavage of the glycosidic bond by C_1_ and C_4_ oxidation, C_6_ oxidation of cellulose has also been reported (19–21) but needs further corroboration.

Currently, fungal LPMOs are classified into five families, AA9, AA11, AA13, AA14, and AA16 (22, 23), and have been shown to be active on cellulose (8, 9, 24, 25), cello-oligosaccharides (18), chitin (26), starch (27), and hemicelluloses such as xyloglucan (12), glucomannan (12), and xylan (28–31). The AA9 family is the second most widely spread LPMO family in fungi after the AA11 family, and dikaryotic fungi with at least one AA9 gene copy have, on average, 12 AA9 LPMO-encoding genes, with some species having more than 50 AA9 gene copies (23). Biochemical characterizations of AA9 LPMOs from the industrially relevant ascomycetes *Myceliophthora thermophila* (32), Neurospora crassa (31, 33), Podospora anserina (34), and *Malbranchea cinnamomea* (30) have provided indications that the multiplicity of AA9s relates to different complex structures in plant cell walls where different biomass polymers are closely associated.

In contrast to traditional GHs, AA9 LPMOs have a flat substrate-binding surface and a solvent-exposed active site, allowing them to bind to crystalline substrates such as cellulose (7, 35). AA9 LPMOs create nicks in recalcitrant regions of the substrate, which results in free chain ends for cellobiohydrolases and endoglucanases to bind to (10, 36, 37). Industrially, AA9 LPMOs have significantly contributed to the recent improvement of cellulolytic enzyme cocktails (38–40) used to saccharify biomass. Moreover, LPMOs have also been used in the production of cellulose nanofibrils (41, 42) and glycoconjugates (43).

**FIG 1:**
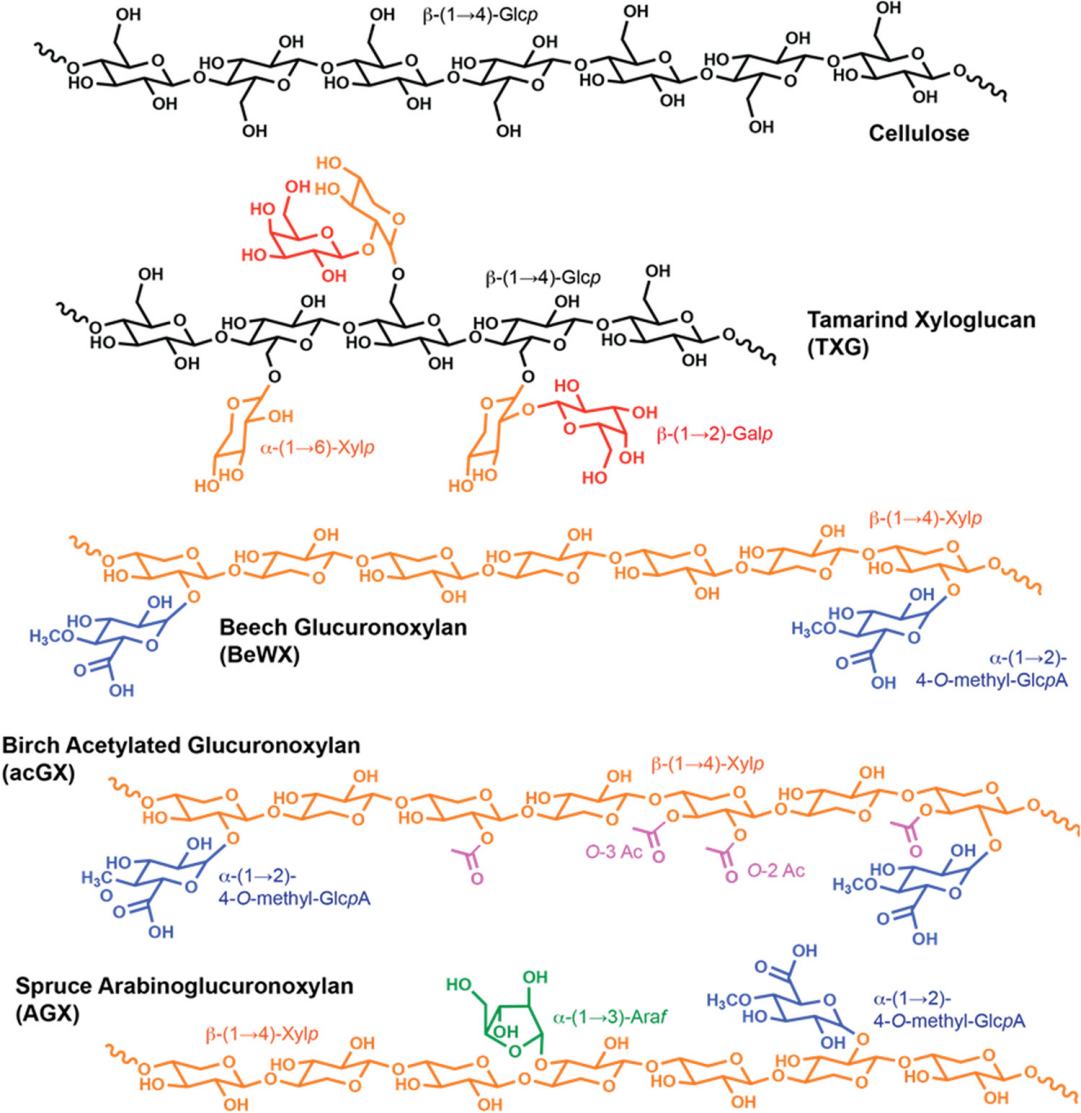
Substrates used in this study. Cellulose is a linear homopolysaccharide comprised of β-(1→4)-glucopyranosyl (Glc*p*) units. Commercial tamarind xyloglucan (TXG) consists of a backbone of β-(1→4)-glucopyranosyl (Glc*p*) units, decorated with α-(1→6)-xylopyranosyl (Xyl*p*) units, which in turn can be further replaced by β-(1→2)-galactopyranosyl (Gal*p*) units. Commercial beech glucuronoxylan consists of a backbone of β-(1→4)-Xyl*p* units decorated with α-(1→2)-methylglucuronopyranosyl (Glc*p*AOMe) units, whereas birch acetylated glucuronoxylan extracted by subcritical water has acetylations of the Xyl*p* backbone in the O_2_ and O_3_ positions (91) in addition to Glc*p*AOMe units. Spruce arabinoglucuronoxylan (AGX) consists of a backbone of β-(1→4)-Xyl*p* units decorated with Glc*p*AOMe units and α-(1→3)-arabinofuranosyl (Ara*f*) units (50).

The composition of lignocellulosic biomass and the molecular structure of its components (mainly cellulose, hemicelluloses, and lignin) can vary significantly between feedstocks, plant species, and tissue type. In this regard, hemicelluloses such as xylans, glucomannans, and β-glucans (e.g., xyloglucan and mixed-linkage β-glucans) comprise a wide variety of linear and branched polysaccharides present in the plant cell wall, interlocking cellulose microfibrils and lignin (44). Hemicelluloses share with cellulose a backbone of β-(1→4)-linked sugar units, but they are often decorated with other glycosyl units and chemically modified by acetylation (Fig. 1). Xyloglucan is the main hemicellulose of the primary cell wall of vascular plants (45). Xylans, on the other hand, are the dominant hemicellulose in hardwood secondary cell walls (mainly acetylated glucuronoxylan) and also occur in the secondary cell walls of softwoods (mainly arabinoglucuronoxylan) (46). Finally, glucomannans are the dominant hemicellulose in softwoods (as acetylated galactoglucomannans), and they are found in minor amounts in hardwoods (as acetylated glucomannans) (47). Hemicelluloses, such as xylans, glucomannans, and xyloglucans, have been reported to adsorb *in vitro* and *in planta* onto the hydrophilic and hydrophobic surfaces of cellulose microfibrils (48–53). For example, xylans can adapt from a 3-fold helical conformation in solution to a 2-fold helical conformation when adsorbing to cellulose microfibrils (48, 50, 54). Illustrating the complexity and variability of these interactions, the adsorption of glucomannans to cellulose depends on the sequence of glucose and mannose sugar units in the backbone (51, 52). The complex interactions between hemicelluloses and cellulose microfibrils and their supramolecular organization in plant cell walls contribute to the recalcitrance of lignocellulosic biomass, which might partially explain the multiplicity of LPMO genes observed in fungal genomes, as different LPMOs may attack physicochemically different parts of the plant cell wall.

To gain more insight into the functional variation among LPMOs produced by a single organism, we used *Thermothielavioides terrestris* (syn. *Thielavia terrestris* [55]), which is an industrially relevant thermophilic ascomycete (56–58). In a study by Berka et al. (56), it was concluded that *T. terrestris* has the enzymatic repertoire necessary to degrade all plant cell wall polysaccharides, and studies on *T. terrestris* secretomes have contributed significantly to the discovery of GH61 enzymes, today known as AA9 LPMOs (59). In our previous studies, we isolated (57), sequenced, and characterized an industrially promising thermophilic strain called *T. terrestris* LPH172 (58). According to bioinformatic annotation, *T. terrestris* LPH172 encoded 18 AA9 LPMOs, five AA11 LPMOs, and one AA16 LPMO. As shown in this previous transcriptomics study, 14 AA9 genes were transcribed during growth on three tested substrates, Avicel, rice straw, and beechwood xylan (58). The high number of AA9 LPMOs and the industrially relevant traits of the strain, such as being able to grow under extreme conditions at 50°C and pH 3 (57), motivated us to characterize AA9 LPMOs from this fungus more comprehensively. We set out to clone, express, and purify LPMOs previously observed to be upregulated by *T. terrestris* LPH172 on Avicel, rice straw, and beechwood xylan (58) to address two key questions: first, does the multiplicity of *T. terrestris* AA9 LPMOs translate to functional differences between the LPMOs, and second, can we identify hitherto unknown specificities of LPMOs on plant polysaccharides?

In the present study, we successfully expressed, purified, and characterized six AA9 LPMOs (including the ortholog of the previously characterized *Tt*LPMO9E, UniProt ID G2RGE5, from *T. terrestris* NRRL 8126) (60). We compared their activities toward both model substrates, process streams and native (hemi)cellulosic substrates, and demonstrated functional variation between the different LPMOs on the tested substrates. Furthermore, we showed that several of these LPMOs harbor novel activities on xylan substrates. We complemented these substrate specificity experiments with comparative studies of the oxidase and peroxidase-like activities of these LPMOs. Our results indicate that the multiplicity of AA9 LPMOs in *T. terrestris* enables the fungus to degrade (physico)chemically different plant cell wall structures.

## RESULTS

### Production of *T. terrestris* LPH172 LPMOs.

Fourteen AA9 LPMOs were found upregulated during *T. terrestris* LPH172 growth on Avicel, rice straw, or beechwood xylan in a previous study (58), all of which contained a signal peptide and were predicted to be secreted. Out of these, six were successfully cloned in P. pastoris and produced at a large scale (nomenclature according to reference 56), with gene identifiers (IDs) according to GenBank assembly GCA_900343105.1 and UniProt ID of the gene orthologs in *T. terrestris* NRRL 8126 (in parentheses): *Tt*LPMO9A (TT08370, G2R6N0), *Tt*LPMO9B (TT04350, G2RB73), *Tt*LPMO9E (TT07456, G2RGE5), *Tt*LPMO9G (TT01736, G2QZK6), *Tt*LPMO9T (TT07455, G2RGE6), and *Tt*LPMO9U (TT04352, G2RB72). The ortholog of the well-studied *Tt*LPMO9E had 100% amino acid sequence identity to the previously studied enzyme from *T. terrestris* NRRL 8126. Figure S1 in the supplemental material shows the amino acid sequences of the six successfully produced *Tt*LPMO9s.

### Sequence and structure analyses.

**FIG 2:**
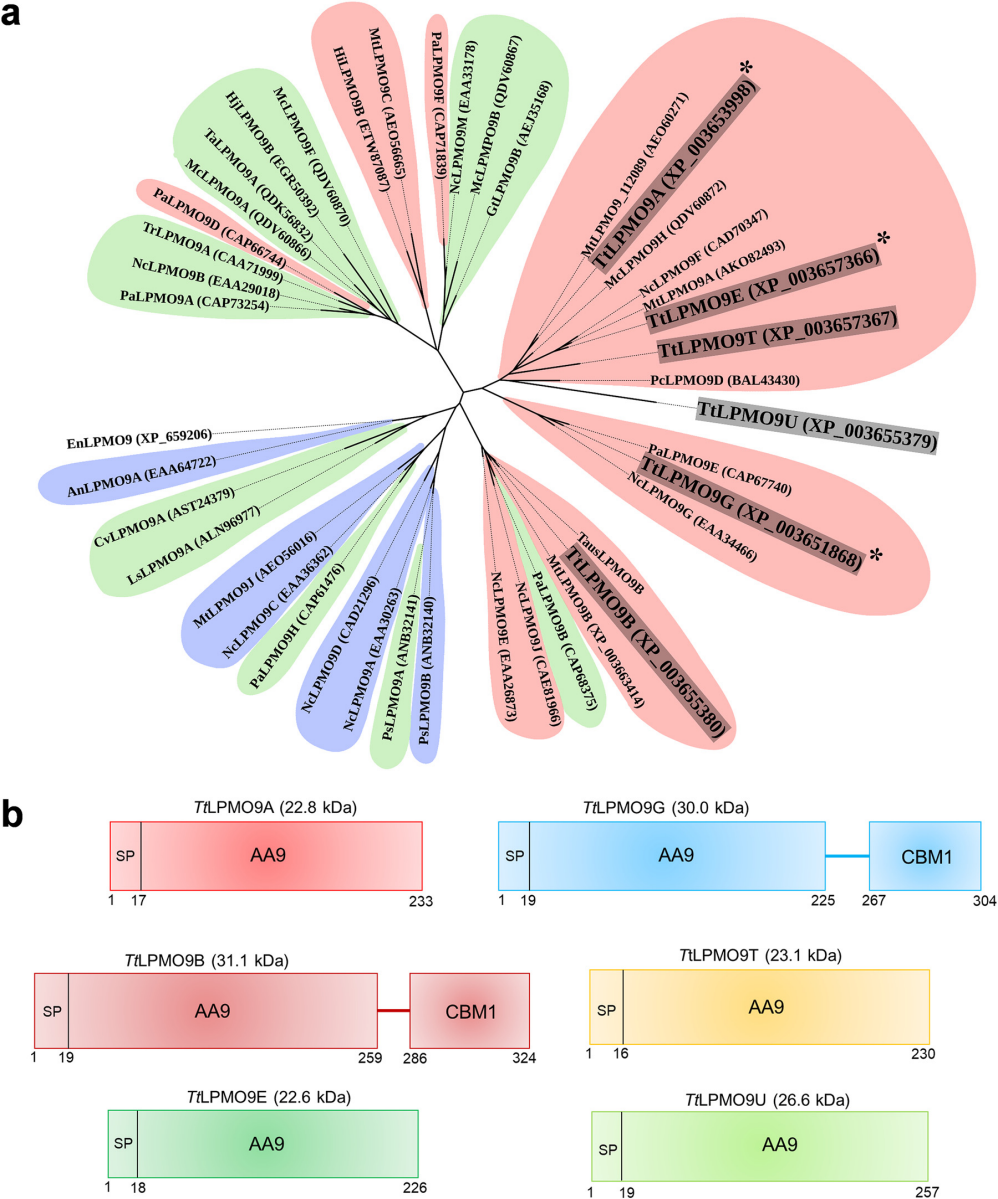
Phylogenetic analysis (a) and modularity of investigated *Tt*LPMO9s (b). (a) An unrooted phylogenetic distance tree of selected characterized LPMOs. The tree shows the evolutionary relationship between the six *Tt*LPMO9s used in this study and 37 previously characterized AA9 LPMOs. The three *Tt*LPMO9s that showed activity toward xylan substrates are marked with an asterisk. The regioselectivity of oxidation on cellulosic substrates are colored: red, C_1_; blue, C_4_; green, C_1_/C_4_. GenBank accession numbers are given in parentheses. (b) Modularity of the tested enzymes. *Tt*LPMO9A, -9E, -9T, and -9U are single-domain AA9s, whereas *Tt*LPMO9B and -9G contain a C-terminal CBM1 domain appended to the AA9 domain. The numbers below the domains represent the amino acid position in the proteins; the size, in parentheses after the LPMO names, indicates the theoretical mass of the full-length proteins, excluding the signal peptide and His_6_ tag (calculated with Expasy’s ProtParam tool).

Phylogenetic analysis of the six *Tt*LPMO9s and 37 characterized LPMO9s revealed that *Tt*LPMO9A, *Tt*LPMO9B, *Tt*LPMO9E, *Tt*LPMO9G, *Tt*LPMO9T, and *Tt*LPMO9U all cluster with C_1_-oxidizing LPMOs (Fig. 2a). The analysis showed that *Tt*LPMO9A clusters more closely with *Mc*LPMO9H from *M. cinnamomea*, *Tt*LPMO9E clusters more closely with N. crassa LPMO9F (*Nc*LPMO9F) and *M. thermophila* LPMO9A (*M*tLPMO9A), while *Tt*LPMO9U clustered more closely with *Pc*LPMO9D from *Phanerochaete chrysosporium*. Interestingly, *Mc*LPMO9H (30), *Nc*LPMO9F (31), and *Mt*LPMO9A (C_1_/C_4_-oxidizing, with preference for C_1_ oxidation) (28) have been shown to oxidatively cleave xylan, while such activity has not been described for any other LPMOs in this cluster. While *Tt*LPMO9A, *Tt*LPMO9E, *Tt*LPMO9T, and *Tt*LPMO9U cluster more closely together, *Tt*LPMO9B and *Tt*LPMO9G are more distantly related to the clade containing the two LPMOs with confirmed activity toward xylan. *Tt*LPMO9G appeared in a clade with *Pa*LPMO9E from *P. anserina* and *Nc*LPMO9G from *N. crassa*, whereas *Tt*LPMO9B clusters further away from the other *Tt*LPMO9s studied here and is more closely related to the C_1_-oxidizing *Mt*LPMO9B from *M. thermophila* (MYCTH_80312), *Nc*LPMO9E and *Nc*LPMO9J from *N. crassa*, and *Thielavia australiensis* LPMO9B (*Taus*LPMO9B) and the C_1_/C_4_-oxidizing *Pa*LPMO9B from *P. anserina*.

Multiple-sequence alignment of the six *Tt*LPMO9s (Fig. S2) showed that all contain the two histidines forming the copper-binding histidine brace, the proximal axial copper-coordinating tyrosine, and the glutamine and histidine in the second coordination sphere (35). *Tt*LPMO9U had two inserts (residues 25 to 37 and 214 to 224) that did not align with any of the other sequences, and *Tt*LPMO9B had an insert from residues 64 to 75. The latter insert is in a region referred to as the L3 loop, and the presence of this loop has been associated with C_4_-oxidizing activity (Fig. S2) (61). In addition, the AA9 domain in *Tt*LPMO9B had an extended C terminus (residues 224 to 240), a feature that has been discussed recently for *Taus*LPMO9B from *T. australiensis* by Calderaro et al. (62). Four of the six studied LPMOs were single-domain enzymes, whereas *Tt*LPMO9B and *Tt*LPMO9G had a C-terminal CBM1 that is attached to the catalytic AA9 domain by a short linker (Fig. 2b).

The crystal structure for *Tt*LPMO9E and homology models of the other five *Tt*LPMO9s built with Phyre2 (http://www.sbg.bio.ic.ac.uk/∼phyre2) showed typical and complete LPMO structures for all six enzymes (Fig. S3a). The models revealed differences in the size and shape of the substrate-binding surfaces and indicate that the two inserts in *Tt*LPMO9U and the single insert in *Tt*LPMO9B affect substrate binding, as they are predicted to be on the binding surfaces of the proteins. Figure S3b provides an identity matrix showing the phylogenetic relationship and sequence identities of the catalytic domains of the six *Tt*LPMO9s and selected AA9 LPMOs.

### Laboratory-scale production.

*Tt*LPMO9A, *Tt*LPMO9B, *Tt*LPMO9G, *Tt*LPMO9T, and *Tt*LPMO9U, all with a C-terminal His_6_ tag, were successfully produced in Pichia pastoris SMD1168H, while *Tt*LPMO9E was produced in P. pastoris PichiaPink strain 4 without a His_6_ tag. Figure S4 shows an SDS-PAGE analysis of the purified proteins. Protein titers varied and were highest for *Tt*LPMO9E. Compared to *Tt*LPMO9E, titers for *Tt*LPMO9A, *Tt*LPMO9B, *Tt*LPMO9G, and *Tt*LPMO9T were lower, and titers for *Tt*LPMO9U were particularly low. For *Tt*LPMO9U, various production parameters were tested to increase the titer. Since adding either 1% (wt/vol) sorbitol or 3% (vol/vol) methanol increased the LPMO yields, 1% (wt/vol) sorbitol was added to all strains except the *Tt*LPMO9E-producing strain. In addition, it was observed that *Tt*LPMO9U disappeared from the supernatant between 72 and 90 h throughout the methanol induction; thus, in the final production the supernatant was harvested after 72 h.

Of all LPMOs studied here, only the catalytic domains of *Tt*LPMO9B and *Tt*LPMO9U contain putative glycosylation sites that might affect substrate binding (Fig. S3a). Glycosylation of LPMOs produced in P. pastoris is common (34, 63), and SDS-PAGE analyses (Fig. S4a) indicated that this also was the case for three of the *Tt*LPMO9s, since these showed higher than expected masses: *Tt*LPMO9B (+17 kDa), *Tt*LPMO9G (+18 kDa), and *Tt*LPMO9U (+10 kDa). *Tt*LPMO9B and *Tt*LPMO9G have a CBM1 domain and a linker, and such linker regions are often heavily O-glycosylated in LPMOs produced in P. pastoris (64). The catalytic domain of *Tt*LPMO9B contains one putative O-glycosylation site that could affect substrate binding (Fig. S3a). Enzymatic deglycosylation of this enzyme was not successful, preventing assessment of the (potential) impact of this (potential) glycosylation. *Tt*LPMO9U is a single-domain LPMO and has four putative N-glycosylation sites (Asn10, Asn52, Asn64, and Asn142), two of which may affect substrate binding (Fig. S3a). De-N-glycosylation of this LPMO was successful (Fig. S4b) but did not affect activity on cellulose (Fig. S4c).

### Oxidase and peroxidase-like activity of *T. terrestris* LPMOs.

H_2_O_2_ production resulting from the oxidase activity of the LPMOs was tested using an assay developed by Kittl et al. (65). This assay couples the reduction of O_2_ and concomitant release of H_2_O_2_ by the LPMO with the oxidation of Amplex Red by horseradish peroxidase. All six LPMO systems produced H_2_O_2_ at rates that exceeded the rate of an enzyme-free copper control reaction, where the LPMO was replaced with CuSO_4_ (Fig. S5a), indicating that all six LPMOs were properly folded and contained a coordinated copper at the active site.

Peroxidase-like activity of the LPMOs was tested by using the 2,6-dimethoxyphenol (2,6-DMP) assay developed by Breslmayr et al. (66, 67). The assay is based on spectrophotometric measurement of coerulignone, which is formed after oxidation of 2,6-DMP by the LPMO, using H_2_O_2_ as a cosubstrate. All six *Tt*LPMO9s oxidized 2,6-DMP to coerulignone (Fig. S5b), which, again, indicated that the LPMOs were properly folded with the copper atom correctly coordinated in the histidine brace.

### Activity on cellulose.

**FIG 3:**
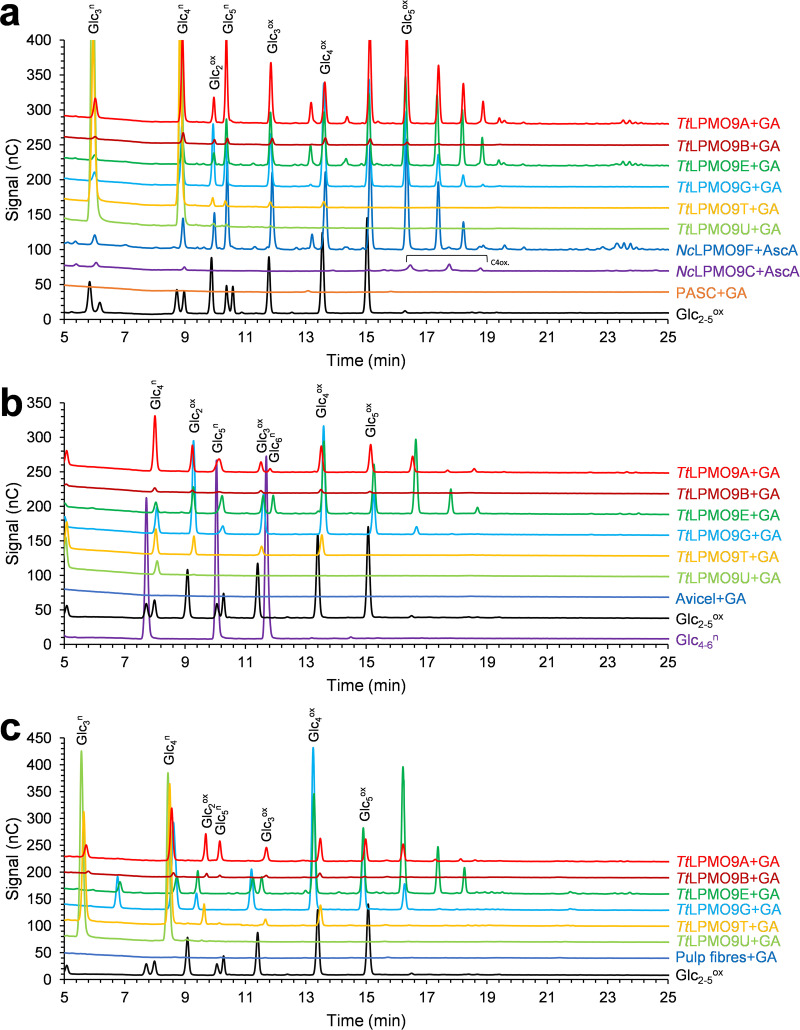
HPAEC-PAD chromatograms showing soluble native and oxidized cello-oligosaccharides released from PASC (a), Avicel (b), or sulfite-pulped spruce fibers (c) by *Tt*LPMO9s. Reaction mixtures contained either 0.4% (wt/vol) PASC, 0.2% (wt/vol) Avicel, or 1% (wt/vol) pulp fibers, 1 μM LPMO in 50 mM BisTris-HCl buffer, pH 6.5, and were incubated at 1,000 rpm and 40°C for 16 h in 100 μL (PASC, Avicel) or 500 μL (pulp fibers). The reactions were started by adding 1 mM reductant (AscA or GA) at time zero. The *Tt*LPMO9s were preincubated with a 0.5 molar equivalent of CuSO_4_ for a minimum of 30 min prior to setting up the reactions. The other LPMOs were copper saturated according to reference 92. Control reactions were performed by replacing the LPMO with 0.5 μM CuSO_4_ (marked as PASC+GA in panel a, Avicel+GA in panel b, or Pulp fibers+GA in panel c) or by replacing the reductant with water (Fig. S6a to c); these reactions did not generate any detectable oxidized products but did generate some native products, which likely are due to contaminating glucanase activity.

Next, all six LPMOs were tested on the following cellulosic substrates: phosphoric acid swollen cellulose (PASC), Avicel, cellopentaose (Glc_5_), and sulfite-pulped spruce fibers (composition is described in Costa et al. [68]). LPMO reaction products were analyzed with high-performance anion-exchange chromatography with pulsed amperometric detection (HPAEC-PAD) for qualitative analysis of general product profiles and with matrix-assisted laser desorption/ionization time-of-flight mass spectrometry (MALDI-TOF MS) for qualitative analysis of possible degradation products. All *Tt*LPMO9 reactions were initiated by the addition of a reductant, gallic acid (GA). HPAEC-PAD analysis revealed reductant-dependent product formation in reactions with PASC by all LPMOs besides *Tt*LPMO9U (Fig. 3a). Interestingly, the enzymes showed considerable variation in apparent product concentrations, with *Tt*LPMO9A, *Tt*LPMO9E, and *Tt*LPMO9G giving the most intense signals. Control reactions without enzyme (Fig. 3a) or reductant (Fig. S6a) showed no production of oxidized cellulose oligomers. The well-studied *Nc*LPMO9F and *Nc*LPMO9C from N. crassa were used as references for C_1_ or C_4_ oxidation, respectively (65). The results revealed that all five *Tt*LPMO9s that were active on PASC most likely generated C_1_-oxidized products only, although generation of small amounts of C_4_-oxidized products cannot be excluded. Closer inspection of Fig. 3a shows minor peaks between the cello-oligosaccharides for the *Tt*LPMO9E reaction, and when analyzed further with MALDI-TOF MS, oxidized and native xylo-oligosaccharides were also detected (Fig. S7), possibly originating from the small amounts of xylan in the PASC substrate. Similar observations were been made previously with *Mt*LPMO9A in reactions with regenerated amorphous cellulose (28) and with *Mc*LPMO9H and *Nc*LPMO9F in reactions with PASC (30, 31). Control reaction mixtures lacking reductant showed production of native cello-oligomers for several LPMO–cellulose combinations. Thus, the native products visible in some of the reactions depicted in Fig. 3 result from a reductant-independent reaction and are likely due to a contaminating glucanase activity. The control reactions (Fig. S6) showed that such glucanase activity was most prominent in *Tt*LPMO9T and *Tt*LPMO9U acting on PASC or spruce fibers, and this is also visible in Fig. 3. Background activities were much lower for the other tested LPMOs (Fig. 3 and Fig. S6). Background activities could affect the product profiles of the LPMOs because initial longer soluble LPMO products may be cleaved by glucanases, and this possibility should certainly be considered for *Tt*LPMO9T. That being said, the profiles of oxidized products depicted in Fig. 3 look like typical product profiles for C_1_-oxidizing LPMOs.

We subsequently investigated the activities of the six LPMOs on Avicel (microcrystalline cellulose) (Fig. 3b) and a more industrially relevant cellulosic substrate, sulfite-pulped spruce fibers containing 87.4% glucan, 7.9% hemicelluloses (2.7% xylan and 5.2% mannan), and 3.3% lignin (68) (Fig. 3c). On these pulp fibers, the highest product signals were observed with *Tt*LPMO9E and *Tt*LPMO9G, followed by *Tt*LPMO9A. It is worth noting the additional low-intensity peaks in the chromatogram for *Tt*LPMO9E, eluting between the oxidized cello-oligosaccharides, which could again be hemicellulose-derived products, as in Fig. 3a. Another noteworthy observation is the apparent low product yields with *Tt*LPMO9A on Avicel (Fig. 3b) and pulp fibers (Fig. 3c) relative to the most active LPMOs, as this difference was not observed on PASC (Fig. 3a). All six *Tt*LPMO9s were also tested for activity on cellopentaose, but no oxidized products were observed for any (data not shown). Although quantitative comparisons of the activities of the various *Tt*LPMO9s on cellulosic substrates is not possible on the basis of the present data, taken together, Fig. 3a to c show that the LPMOs have different preferences when it comes to the analyzed cellulosic substrates.

### Activity on hemicelluloses.

The six *Tt*LPMO9s were tested in reactions with the following hemicellulosic substrates: tamarind xyloglucan (TXG), beechwood xylan (BeWX), spruce arabinoglucuronoxylan (AGX), acetylated glucuronoxylan from birch (acGX), konjac glucomannan (KGM), wheat arabinoxylan (WAX), and acetylated galactoglucomannan from spruce (acGGM). The substrates were tested either alone or in mixture with PASC. Reactions were set up as described previously, with 1 μM LPMO in 50 mM BisTris-HCl buffer, pH 6.5, and were initiated by the addition of 1 mM reductant (GA).

**FIG 4:**
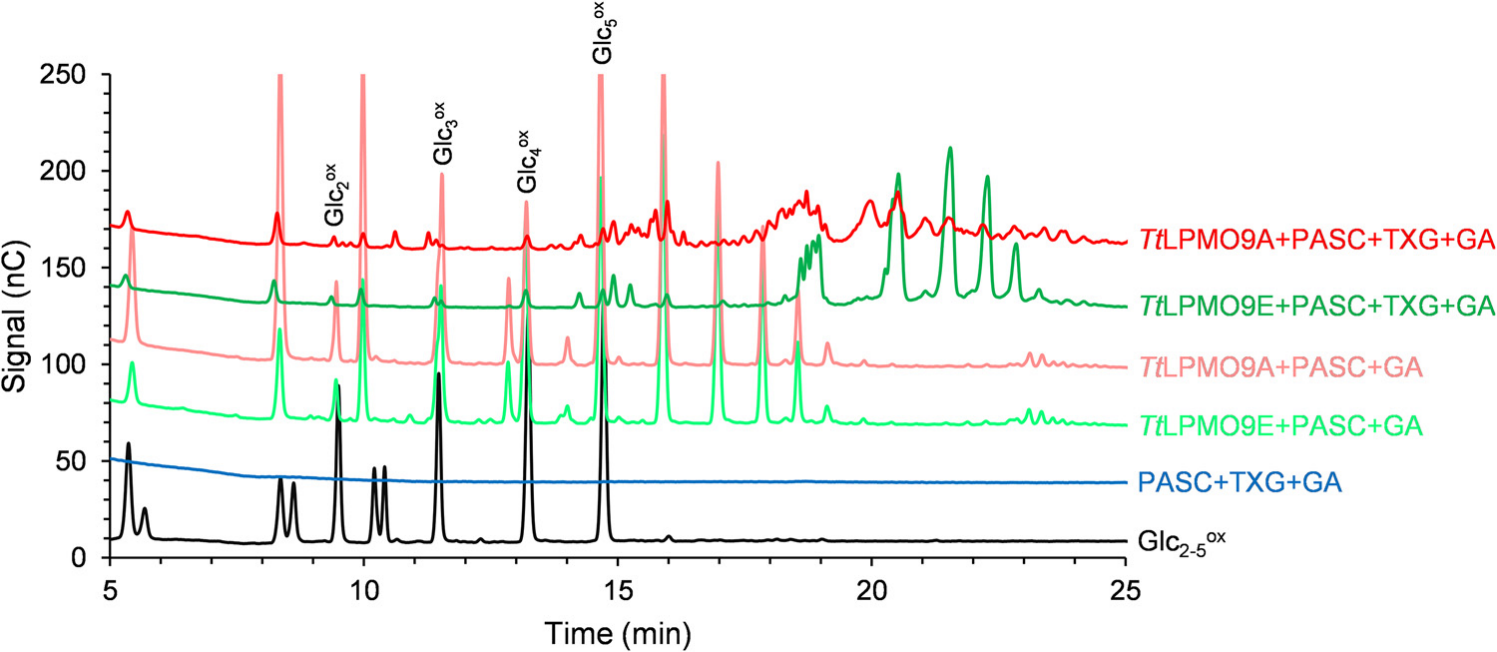
HPAEC-PAD chromatograms showing soluble native and oxidized oligosaccharides released from PASC and TXG by *Tt*LPMO9A and *Tt*LPMO9E. The reaction mixtures contained 0.4% (wt/vol) PASC or 0.2% (wt/vol) PASC plus 0.2% (wt/vol) TXG and 1 μM LPMO in 50 mM BisTris-HCl buffer, pH 6.5, and were incubated at 1,000 rpm and 40°C for 16 h in 100 μL. The reactions were initiated by adding 1 mM reductant (GA) at time zero. The *Tt*LPMO9s were preincubated with a 0.5 molar equivalent of CuSO_4_ a minimum for 30 min before setting up the reaction. Control reactions were performed by replacing LPMO with 0.5 μM CuSO_4_ (marked PASC+TXG+GA) or by replacing reductant with water (Fig. S8a) and did not generate any detectable oxidized products. Similar reactions with the other *Tt*LPMO9s showed little or no TXG-derived product (Fig. S8b). Figure S8c shows reductant-dependent product formation in reactions with only TXG, which was absent from or very low for all LPMOs.

**FIG 5:**
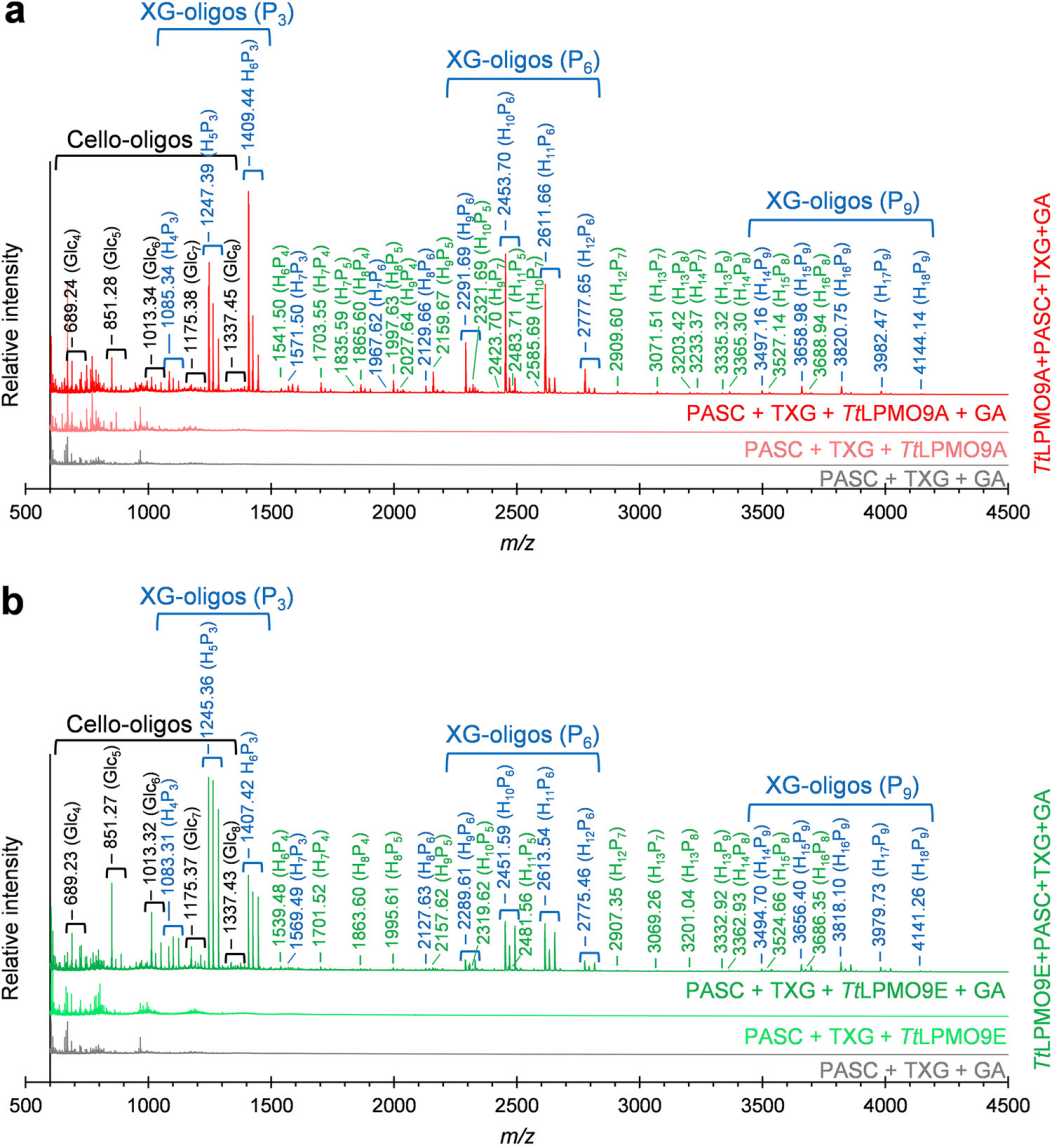
MALDI-TOF MS spectra of TXG cleavage pattern differences of *Tt*LPMO9A (a) and *Tt*LPMO9E (b). The reaction mixtures contained 0.4% (wt/vol) PASC or 0.2% (wt/vol) PASC plus 0.2% (wt/vol) TXG and 1 μM LPMO in 50 mM BisTris-HCl buffer, pH 6.5, and were incubated at 1,000 rpm and 40°C for 16 h in 100 μL. The reactions were initiated by adding 1 mM reductant (GA) at time zero. The *Tt*LPMO9s were preincubated with a 0.5 molar equivalent of CuSO_4_ a minimum of 30 min prior to setting up the reaction. Control reactions were performed by replacing LPMO with 0.5 μM CuSO_4_ (marked PASC+TXG+GA) or by replacing reductant with water (marked PASC+TXG+LPMO) and did not generate any detectable oxidized products. In both panels, the Na^+^ adducts of solubilized cello-oligosaccharides (native; the most dominant species for both LPMOs) and XG-oligosaccharides (native for *Tt*LPMO9A and hydrated oxidized for *Tt*LPMO9E) are indicated. XG-oligosaccharides are annotated according to the number of hexose (H) and pentose (P) units. XG-oligosaccharides with 3*n* pentose units are labeled in blue, while other numbers of pentose units are in green.

**FIG 6:**
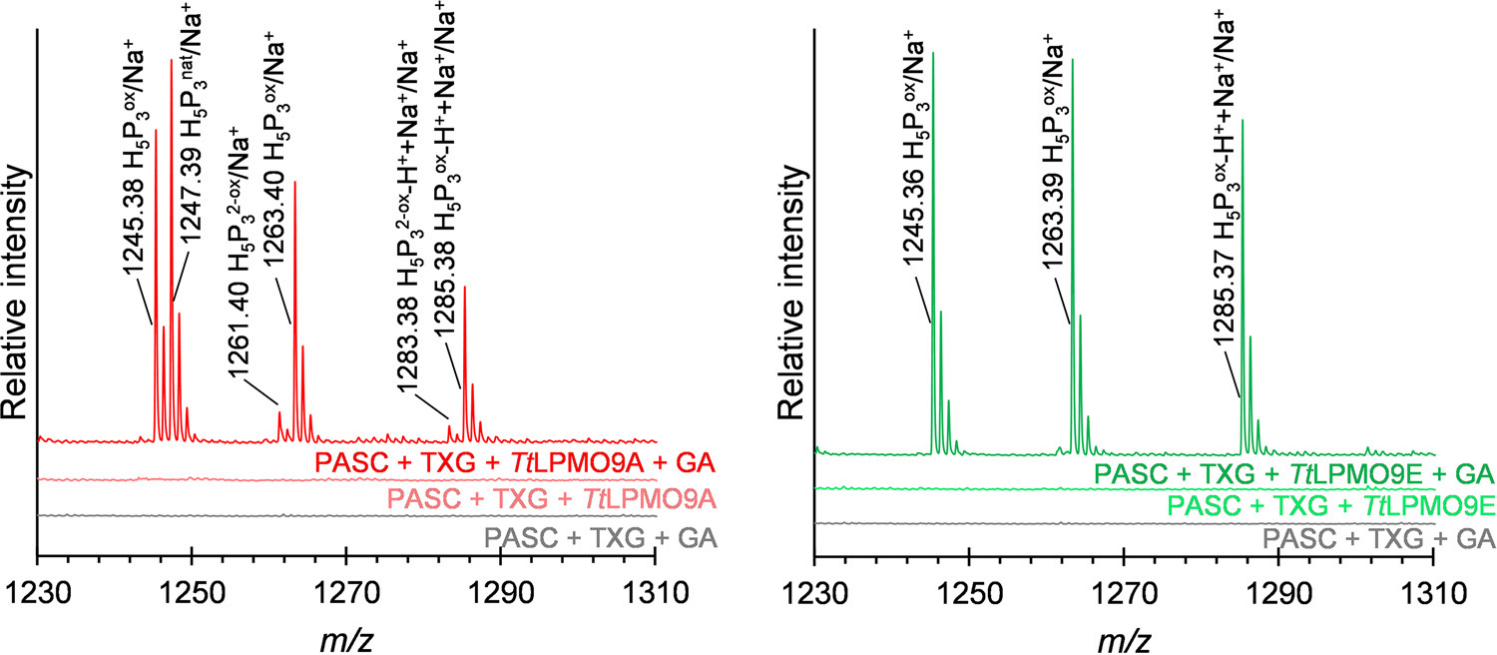
MALDI-TOF MS analysis showing the difference in regioselectivity of *Tt*LPMO9A (left) and *Tt*LPMO9E (right) on xyloglucan. Shown is a zoom-in on the H_5_P_3_ cluster shown in Fig. 5a and b. The Na^+^ adducts of XG-oligosaccharides are labeled. Oxidized oligosaccharides are marked ox, and double oxidation (for *Tt*LPMO9A) is marked 2-ox. The Na^+^ salt of the (C_1_- or C_1_/C_4_-) oxidized oligosaccharides are marked -H^+^+Na^+^.

Reactions with TXG and PASC showed that both *Tt*LPMO9A and *Tt*LPMO9E were clearly active on xyloglucan (Fig. 4; see also Fig. 6), while *Tt*LPMO9G and *Tt*LPMO9T had detectable but low TXG activity (Fig. S8b). When comparing the chromatograms for reactions of *Tt*LPMO9A and *Tt*LPMO9E with a mixture of TXG and PASC with those for reactions with PASC alone (Fig. 4), we observed reductant-dependent formation of several novel products, clearly demonstrating that these two enzymes can oxidize TXG. When TXG was the only substrate present, these LPMOs showed drastically reduced product levels (Fig. S8c), indicating that PASC was needed to make the LPMOs bind productively to the TXG substrate. Interestingly, the profiles of the TXG-derived products differed between *Tt*LPMO9A and *Tt*LPMO9E (14 to 23 min in Fig. 4). Reactions with *Tt*LPMO9E resulted in much fewer products than *Tt*LPMO9A, which, based on analogy with previous studies on TXG-active LPMOs, indicates a difference in cleavage preference depending on substitutions of the glucan (12, 63). To confirm that the apparent differences in product profiles obtained in reactions with TXG and PASC mixtures were not merely due to differences in the extent of reactions, the samples were analyzed with MALDI-TOF MS to get better insight into the nature of the products (Fig. 5 and 6). Most of the products in reactions with *Tt*LPMO9E contained multiples of three pentose units (3*n*), whereas *Tt*LPMO9A generated a range of XG-oligosaccharides with more even distribution of numbers of pentose units. This suggests that *Tt*LPMO9E is a little tolerant to substitution adjacent to the scissile bond, while *Tt*LPMO9A is tolerant to substitution adjacent to the scissile bond, a type of action that has been described for C_1_/C_4_-oxidizing LPMOs (69). Notably, the most dominant species were the oxidized species for *Tt*LPMO9E and the native species for *Tt*LPMO9A (which were labeled accordingly in Fig. 5a and b). A closer look at the H_5_P_3_ cluster, which is representative of the complete spectrum, revealed that *Tt*LPMO9E generated only oxidized XG-oligosaccharides that are consistent with C_1_ oxidation, whereas *Tt*LPMO9A generated native, oxidized, and double-oxidized products, which indicate that, on xyloglucan, this enzyme catalyzes C_4_ oxidation (Fig. 6). Thus, our data indicate that the apparent difference in XG-derived product profiles is the result of a difference in both cleavage pattern and oxidative regioselectivity, the latter of which, to our knowledge, has not been described previously. It is worth noting, and not without precedent (70), that our data indicate that the oxidative regioselectivity of *Tt*LPMO9A (likely C_1_ on cellulose and C_1_/C_4_ on TXG) is substrate dependent.

Xyloglucan-active LPMO9s have been studied in quite some detail, and several authors have tried to link sequence features of the LPMOs (in particular the presence and length of certain loop regions) to observed XG activity and the impact of substitutions (69, 71, 72). Interestingly, the observed activities of *Tt*LPMO9A and *Tt*LPMO9E do not match with these previous classifications, which would have predicted these two LPMOs to be inactive on TXG, or, when active, to both be substitution tolerant.

**FIG 7:**
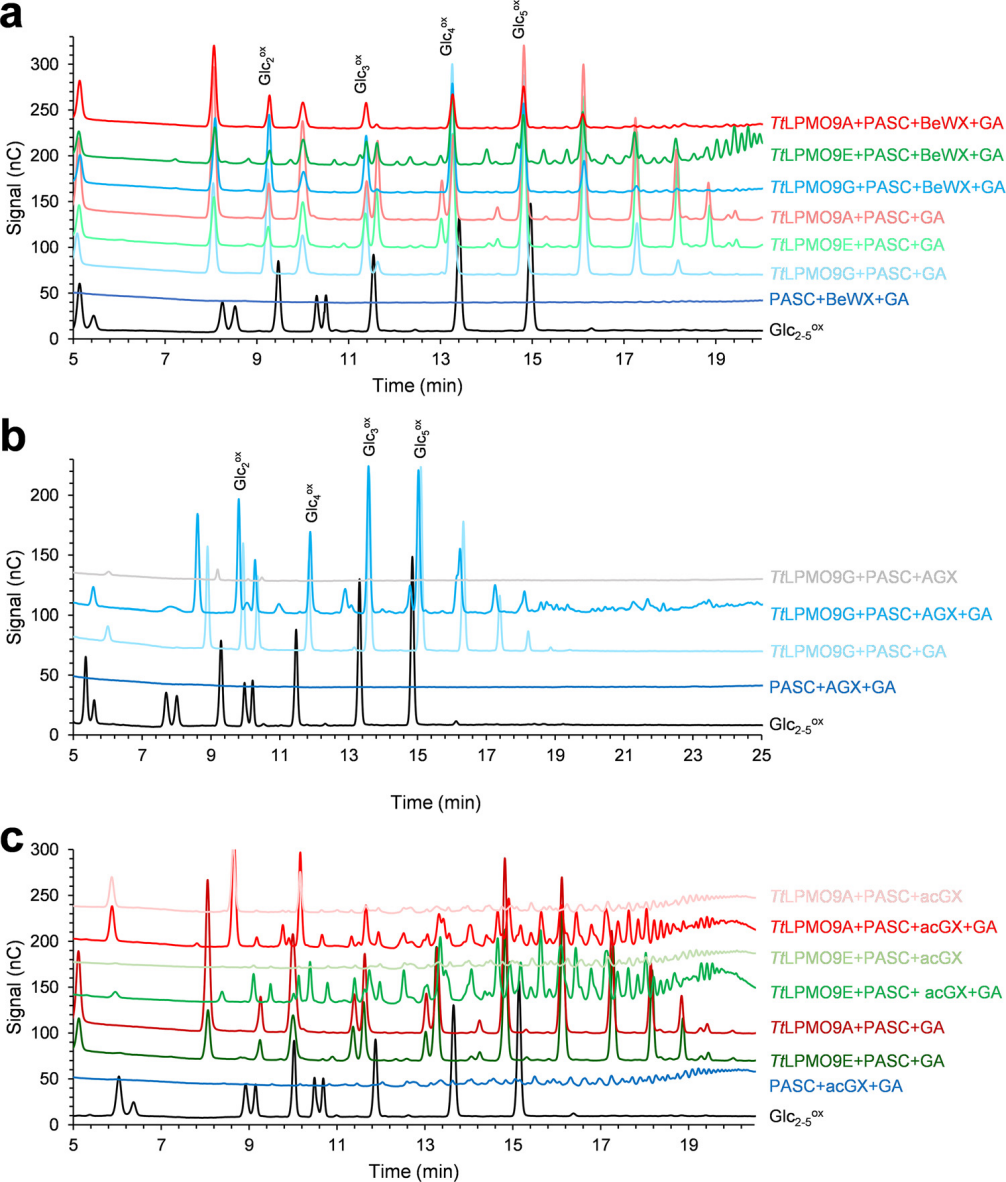
HPAEC-PAD chromatograms of soluble products generated upon incubating a mixture of PASC and BeWX with *Tt*LPMO9A, -9E, and -9G (a), PASC and AGX with *Tt*LPMO9G (b), and PASC and acGX with *Tt*LPMO9A and -9E (c). Reaction mixtures contained either 0.4% (wt/vol) PASC or 0.2% (wt/vol) PASC with 0.2% (wt/vol) xylan, 1 μM LPMO, in 50 mM BisTris-HCl buffer, pH 6.5, and were incubated at 1,000 rpm and 40°C for 16 h in 100 μL. The reactions were started by adding 1 mM reductant (GA) at time zero. The *Tt*LPMO9s were preincubated with a 0.5 molar equivalent of CuSO_4_ for a minimum of 30 min prior to setting up the reactions. Control reactions were performed by replacing the LPMO with 0.5 μM CuSO_4_ (marked as PASC+xylan+GA) or by replacing the reductant with water (see Fig. S9a for PASC+BeWX reactions; control reactions for PASX+AGX and PASC+acGX reactions are shown in panels b and c); these reactions did not generate any detectable oxidized products but did generate some native products, which likely are due to contaminating glucanase activity. Figure S9b to d shows reductant-dependent product formation in reactions with only BeWX, AGX, or acGX, respectively; none of these reactions showed product formation except for trace amounts of products in the reaction of *Tt*LPMO9G with AGX.

While activity of AA9 LPMOs on hemicelluloses with β-(1→4)-glucan backbones (e.g., TXG, GGM, and KGM) is more common (12, 30, 33, 63), activity toward xylans has been demonstrated only in a few cases (28, 30, 31). Interestingly, HPAEC-PAD analysis of reaction mixtures containing both PASC and BeWX showed formation of non-cellulose-derived products for the reactions with *Tt*LPMO9A, *Tt*LPMO9E, and *Tt*LPMO9G (Fig. 7a). These additional products were not observed in control reactions without reductant (Fig. S9a) or in reactions containing only PASC (Fig. 7a) or only BeWX (Fig. S9b).

**FIG 8:**
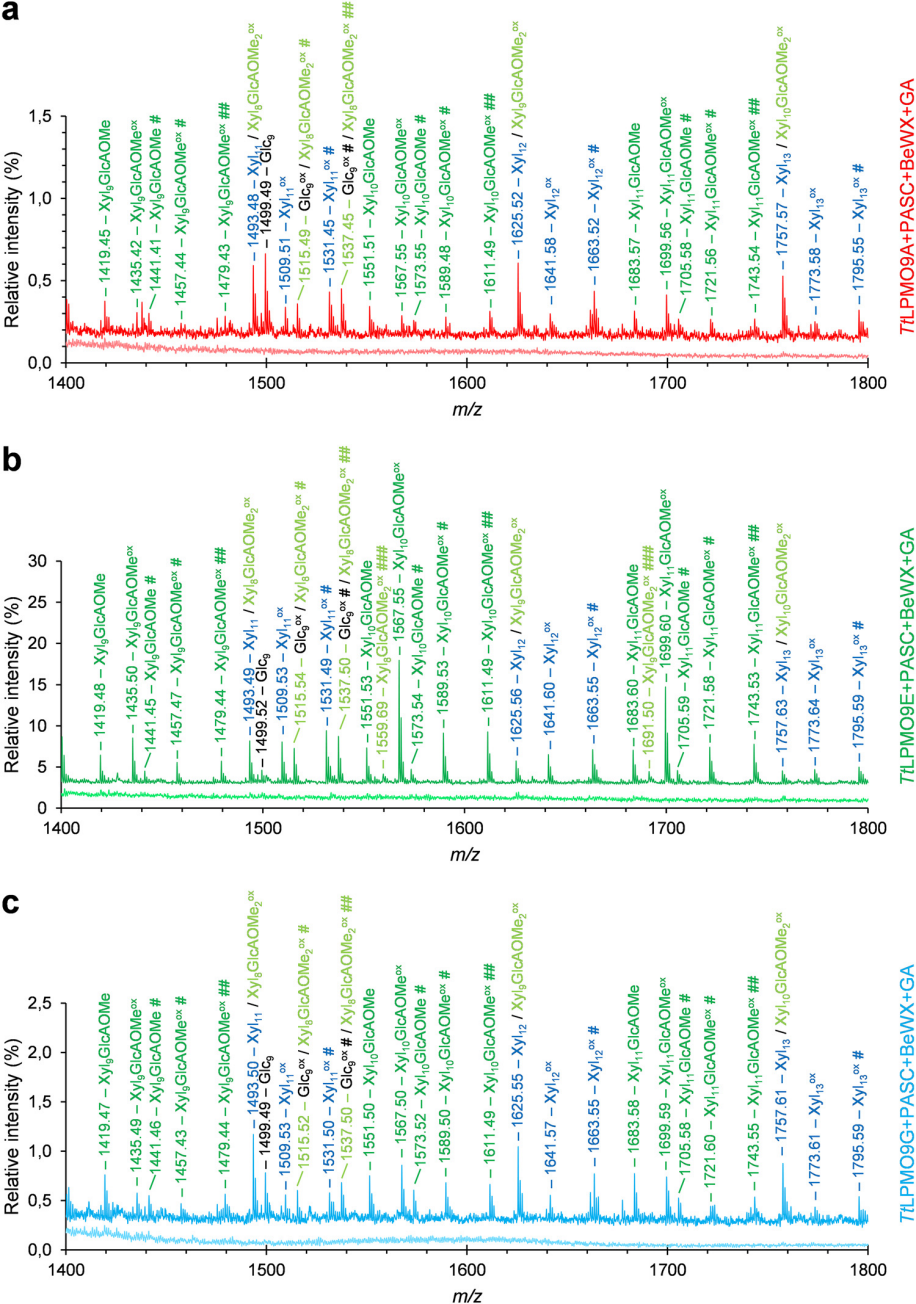
MALDI-TOF MS spectra of products generated by *Tt*LPMO9A (a), *Tt*LPMO9E (b), and *Tt*LPMO9G (c) in PASC and BeWX mixtures. The reaction mixtures contained 0.2% (wt/vol) PASC with 0.2% (wt/vol) BeWX, 1 μM LPMO in 50 mM BisTris-HCl buffer, pH 6.5, and were incubated at 1,000 rpm and 40°C for 16 h in 100 μL. The reactions were started by adding 1 mM reductant (GA) at time zero. The *Tt*LPMO9s were preincubated with a 0.5 molar equivalent of CuSO_4_ for a minimum of 30 min before setting up the reactions. Spectra of the reactions with (dark colors) and without (light colors) GA are shown; the signal intensities are shown relative to the intensity of the cello-oligosaccharide signal with the highest intensity for each spectrum. All labeled peaks are sodium adducts. Sodium salts (+22 per sodium), which can be formed through binding to free carboxylic groups in Glc1A and Xyl1A (generated by C_1_ oxidation) and/or in GlcAOMe substitutions, are annotated with # or ## for one or two Na^+^ ions, respectively. C_1_-oxidized products are labeled ox. Glucuronoxylan oligomers with one or two GlcAOMe substitutions are labeled in dark or light green, respectively. Nonsubstituted xylo-oligomers are labeled in blue, while cello-oligomers are labeled in black. Reactions with the other three *Tt*LPMO9s (data not shown) or with BeWX alone (Fig. S9b) did not yield detectable amounts of products.

To confirm that these LPMOs indeed degraded xylan and generated oxidized xylo-oligosaccharides, we further analyzed the same samples with MALDI-TOF MS. As predicted, we observed several products with masses corresponding to oxidized xylo-oligosaccharides for *Tt*LPMO9A, *Tt*LPMO9E, and *Tt*LPMO9G; these products included both linear (in blue) and 4-*O*-methyl-d-glucuronic acid (GlcAOMe)-substituted (single substitution, in dark green; double substitution, in light green) xylo-oligosaccharides, as shown for the *m/z *= 1,400 to 1,800 range in Fig. 8a to c. The spectra for control reactions without reductant are indicated below main spectra and show no oxidized or native products. Notably, the relative intensities between the highest xylan- and cellulose-derived signals (ca. 0.5%, 13%, and 1.0% for *Tt*LPMO9A, -9E, and -9G, respectively) in the MALDI-TOF MS spectra (see also the extended spectrum in Fig. S10 with a broader product range, *m/z *= 600 to 2,600, also showing the cello-oligosaccharide peaks with the highest intensity) were well aligned with the relative difference in the size of the peaks attributed to cello-oligosaccharide peaks and the unknown, potentially xylan-derived oligosaccharides in the HPAEC-PAD chromatograms shown in Fig. 7a.

Following the observation that three of the six tested *Tt*LPMO9s were active on BeWX, we also tested their activity toward two additional xylan substrates in combination with PASC: spruce arabinoglucuronoxylan (AGX) and birch acetylated glucuronoxylan (acGX). Reactions were set up under the same conditions as those for BeWX. In the reactions with AGX, interestingly, only *Tt*LPMO9G showed reductant-dependent activity (Fig. 7b). We observed a range of novel peaks eluting between 19 and 25 min, in addition to the cellulose-derived products in the control reactions with only PASC. Despite the clear signals of unknown, potentially xylan-derived products in the HPAEC-PAD chromatograms, however, we were not able to detect oxidized xylan-derived products by *Tt*LPMO9G on AGX combined with PASC using MALDI-TOF MS. Interestingly, reactions with *Tt*LPMO9G and AGX alone showed low levels of reductant-dependent product formation (Fig. S9c), indicating that, in contrast to all other tested *Tt*LPMO–xylan combinations (Fig. S9b to d), this LPMO has some activity on non-cellulose-bound AGX.

**FIG 9:**
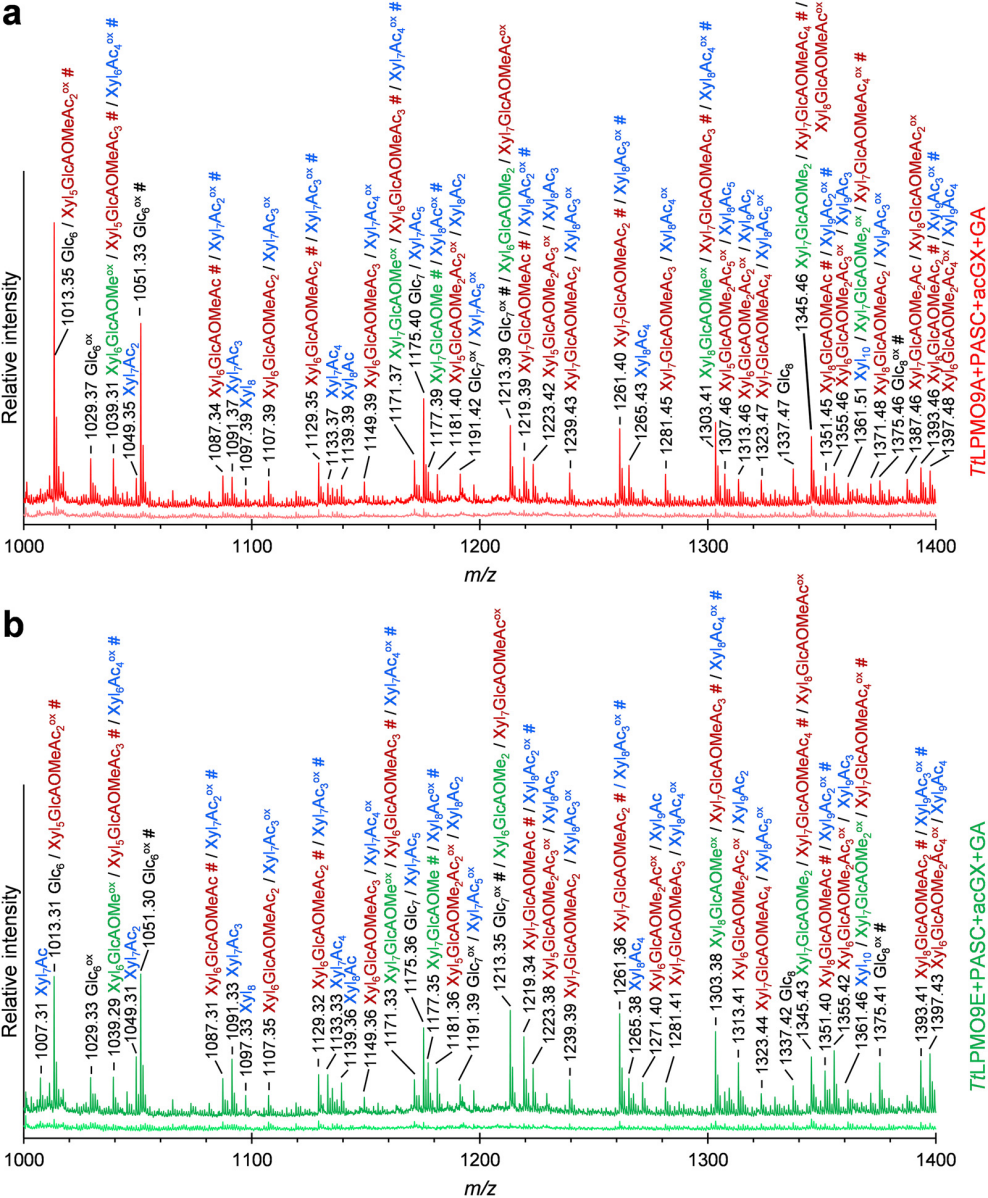
MALDI-TOF MS spectra of products generated by *Tt*LPMO9A (a) and *Tt*LPMO9E (b) in PASC and acGX mixtures. The reaction mixtures contained 0.2% (wt/vol) PASC, 0.2% (wt/vol) acGX, and 1 μM LPMO in 50 mM BisTris-HCl buffer, pH 6.5, and were incubated at 1,000 rpm and 40°C for 16 h in 100 μL. The reactions were started by adding 1 mM reductant (GA) at time zero. The *Tt*LPMO9s were preincubated with a 0.5 molar equivalent of CuSO_4_ for a minimum of 30 min before setting up the reaction. Spectra of the reactions with (dark colors) and without (light colors) GA are shown. All labeled peaks are sodium adducts. Sodium salts (+22 per sodium), which can be formed through binding to free carboxyl groups in the terminal Glc1A or Xyl1A and in the GlcAOMe substitutions, are annotated with # or ## for one or two Na^+^ ions, respectively. C_1_-oxidized products are labeled ox. Cellulose-derived products are labeled in black; nonglucuronylated but acetylated acGX-derived products are labeled in blue; glucuronylated acGX-derived products are labeled in red (acetylated) and green (nonacetylated). Because of the heterogeneous nature and high complexity of the substrate, the majority of the identified masses correspond to several (oxidized and nonoxidized) oligosaccharides with the same overall composition or mass but different structure. Reactions with the other four *Tt*LPMO9s on PASC and acGX mixture did not yield detectable amounts of products (data not shown).

In contrast to the mixture of PASC and AGX, *Tt*LPMO9G showed no activity on the mixture of PASC and acGX (data not shown). On the other hand, *Tt*LPMO9A and *Tt*LPMO9E showed clear reductant-dependent activity on PASC and acGX (but not on acGX alone; Fig. S9d). We observed several novel products with HPAEC-PAD (Fig. 7c), and further analyses with MALDI-TOF MS revealed products with masses possibly belonging to a variety of native and oxidized xylo-oligosaccharides, both with and without 4-*O*-methylglucuronylation and/or acetylation (Fig. 9). Some caution is needed when interpreting these results, since acGX is a heterogeneous substrate; thus, the detected masses can represent several oxidized and nonoxidized cellulose and xylan degradation products with the same overall mass and/or composition but different structure.

Table 1 shows a summary of the detected activities for the six tested *Tt*LPMO9s.

**TABLE 1:** Summary of the activities of the six *Tt*LPMO9s on the 15 tested substrates and substrate combinations, as derived from HPAEC chromatograms of product profiles^*a*^

Substrate	*Tt*LPMO9A	*Tt*LPMO9B	*Tt*LPMO9E	*Tt*LPMO9G	*Tt*LPMO9T	*Tt*LPMO9U
PASC	+++	+	+++	+++	+	–
Avicel	++	+	+++	+++	+	–
Pulp fibers	++	+	+++	+++	+	–
Glc_5_	–	–	–	–	–	–
PASC+KGM	–	–	–	–	–	–
PASC+acGGM	–	–	–	–	–	–
PASC+WAX	–	–	–	–	–	–
PASC+TXG	+++	–	+++	+	(+)	–
TXG	(+)	–	(+)	(+)	(+)	–
PASC+BeWX	+	–	+++	+	–	–
BeWX	–	–	–	–	–	–
PASC+AGX	–	–	–	++	–	–
AGX	–	–	–	(+)	–	–
PASX+acGX	+++	–	+++	–	–	–
acGX	–	–	–	–	–	–

a Activity is indicated: (+), minor (trace activity); +, low; ++, medium; +++, high; –, absent. These are rough, not truly quantitative assessments that give an overall impression of the variation in substrate specificity. For each substrate, +++ stands for the maximum observed product level for that substrate.

## DISCUSSION

In the present study, we functionally characterized six AA9 LPMOs from the thermophilic fungus *T. terrestris* LPH172 and found that five of them cleave cellulosic substrates by oxidation at the C_1_ position, with clear differences between the LPMOs over different types of cellulosic substrates. Studies with hemicellulose–cellulose mixtures revealed additional and major functional differences between the LPMOs, related to the ability to cleave various types of xylan. Of note, this study also revealed hitherto undetected catalytic capabilities of one of the earliest characterized and most studied AA9 LPMOs, *Tt*LPMO9E (60), highlighting the complexity of assessing LPMO substrate specificity. These results underline that the multiplicity of the AA9 LPMO genes in filamentous fungi relates to variation in the copolymeric polysaccharide structures present in plant cell walls, as suggested previously (30, 32–34).

To the best of our knowledge, LPMO activities on spruce arabinoglucuronoxylan and birch acetylated glucuronoxylan have not been reported previously. Importantly, these substrates are likely much closer to natural substrates than previously used model xylan substrates. Variation in the xylan structure clearly influenced the activity of the tested LPMOs. The fact that the three xylan-active LPMOs identified in this study (namely, *Tt*LPMO9A, *Tt*LPMO9E, and *Tt*LPMO9G) show distinct preferences toward various xylans shows the importance of including a large variation of substrates in functional screening of LPMOs.

The possible interplay between the xylan-active LPMOs and the chemical structure of the xylans and other hemicelluloses, in terms of the degree, pattern, and chemical nature of the substitutions, which all influence xylan binding onto cellulose (48, 50, 73–76), may also affect the interaction with the LPMO's substrate-binding surface. For example, it is well known that binding of xylan onto cellulose is promoted by an even pattern of the substitutions, leaving the side of the molecule that is void of substitutions ready to interact with the surface of cellulose microfibrils (75). Our results suggest that the occurrence of a repetitive pattern of substitution in xylans, as is the case for BeWX, AGX, and acGX, promotes *Tt*LPMO9 activity on xylan. Unraveling the structural determinants of the different substrate specificities of *Tt*LPMO9A, -9E, and -9G on xylans is an interesting topic for further studies of these LPMOs.

Interestingly, none of the tested LPMOs had any activity on wheat arabinoxylan, cellopentaose, konjac glucomannan, or acetylated galactoglucomannan from spruce. For example, a study of four AA9 LPMOs from the thermophilic fungus *M. cinnamomea* showed that two of these had activity on cellohexaose (*Mc*LPMO9A and *Mc*LPMO9F, both C_1_/C_4_-oxidizing), whereas one was also active on konjac glucomannan (*Mc*LPMO9A, C_1_/C_4_-oxidizing) (30). Similar results have been obtained for N. crassa AA9 LPMOs, of which *Nc*LPMO9C (C_4_-oxidizing) was active on cello-oligosaccharides and glucomannan (33). However, it must be noted that five of the six tested *T. terrestris* LPMO9s cluster closely together in our phylogenetic analysis (Fig. 2a) and that all of them are predominantly C_1_-oxidizing on cellulose. To date, no C_1_-oxidizing LPMOs have been shown to be active on soluble cello-oligosaccharides. While this subset of upregulated *Tt*LPMO9s clearly has the ability to act on complex biomass, covering quite a few substrates, the other 12 *Tt*LPMO9s present in the *T. terrestris* LPH172 genome (also) may act on additional substrates. Curiously, *Tt*LPMO9U was not active on any of the tested substrates, even though it was active in the H_2_O_2_ production and H_2_O_2_ consumption assays (Fig. S5a and b). As shown in Fig. S2 and S3, the enzyme has two loop inserts near the substrate binding surface that are not present in the other tested LPMOs. These loops could either hinder binding of substrates or promote binding of substrates that were not tested in our study.

Both the choice and concentration of reducing agent are known to have major impacts on LPMO kinetics (77–79). In the present study, we have primarily used gallic acid to reduce the LPMOs’ active-site copper, since it has been reported to result in more stable reactions with AA9s, ultimately leading to higher product titers (78). Ascorbic acid, which is the most commonly used reductant in LPMO experiments, generally results in faster kinetics than GA but also results in faster inactivation of the LPMO due to potentially excessive H_2_O_2_ production, although this is very dependent on the conditions used and on the LPMO (79). It is possible that the redox potentials of the active-site coppers of the LPMOs differ significantly, which in turn could result in different reduction and H_2_O_2_ generation kinetics. Furthermore, it is possible that other reductants that have not yet been tested with *Tt*LPMO9s will give different results, including higher product yields or even different product compositions or detection of hitherto undetected activities. Possible redox partner candidates would be glucose–methanol–choline (GMC) oxidoreductases in the AA3_1 and AA3_2 subfamilies, which were also shown to be coexpressed and co-upregulated together with the AA9 LPMOs in our previous transcriptome study (58). Future studies of externally supplied H_2_O_2_ are also likely to broaden our understanding of functional differences between the *Tt*LPMO9s.

It remains unknown if and how LPMOs with hemicellulolytic activities boost the efficiency of cellulolytic enzyme cocktails acting on xylan-containing substrates beyond what can be achieved with cellulose-active LPMOs. Studies on a xylan-active AA14 LPMO have indicated that this may be the case (29). It would be interesting to explore whether the boost in cellulose hydrolysis by *Tt*LPMO9E, as observed by Harris et al. (60), can be related to the xylanolytic ability of this LPMO, which was unknown at the time. This is quite plausible, since the substrate used, pretreated corn stover, contained 7.0% (wt/wt) xylan, the (more efficient) removal of which could increase the access to cellulose for cellulases and cellulose-active LPMOs. Furthermore, the possible increase in pentose monomer concentrations due to xylanolytic LPMO activity could mean an increase in the yields of fermentable sugars and, consequently, a potential increase in fermentation product yields in biorefineries.

In conclusion, our results revealed unprecedented LPMO activities on xylan and show that *T. terrestris* LPMOs have evolved to act on different parts of lignocellulosic biomass. By secreting promiscuous LPMOs that attack both cellulosic and hemicellulosic compounds, the fungus likely maximizes substrate utilization, which would provide a clear fitness advantage.

## MATERIALS AND METHODS

### Structure and sequence analyses.

Multiple-sequence alignment of *Tt*LPMO9s was performed with Clustal Omega (https://www.ebi.ac.uk/Tools/msa/clustalo/), and the obtained results were edited with JalView (https://www.jalview.org/). Homology modeling was done using the Phyre2 online tool (http://www.sbg.bio.ic.ac.uk/∼phyre2) (80), using only the LPMO domains and removing the signal peptides. The template structures were *Nc*LPMO9F (PDB entry 4QI8) for *Tt*LPMO9A, -9T, and -9G and *Nc*LPMO9C (4D7U) for *Tt*LPMO9B and *Tt*LPMO9U. PyMOL (The PyMOL Molecular Graphics System, version 0.99, Schrödinger, LLC) was used for analyzing the models. For phylogenetic and sequence analysis, the sequences of *Tt*LPMO9A, -9B, -9E, -9G, -9T, and -9U were aligned with 37 previously characterized LPMO9s using the online T-Coffee tool (http://tcoffee.crg.cat/apps/tcoffee/do:regular) (81). The sequences were trimmed of signal peptides, and only the AA9 domains of the sequences were used. The resulting multiple-sequence alignment was edited with AliView 1.27 (82). Phylogenetic analysis was performed using the ProtTest 3.4 software package (83), using the default settings to compute likelihood scores (including all substitution matrices), and a consensus tree was then built using the Akaike information criterion and a majority-rule consensus type. The resulting consensus tree was edited for publication using iTol v6 (https://itol.embl.de/) (84).

### Gene cloning of *Tt*LPMO9A, -9B, -9G, -9T, and -9U.

The coding sequences of the five genes were retrieved from the sequenced *T. terrestris* LPH172 (GenBank assembly accession number GCA_900343105.1), and the LPMOs were named according to Berka et al. (56). The amino acid sequences are found in Fig. S1 in the supplemental material. Codon-optimized coding sequences for heterologous expression in P. pastoris were ordered from Twist Bioscience (San Francisco, CA, USA) and contained correct restriction sites and native signal peptides. Primers Tt9G-F (5′→3′, GCGGCGTTCGAAACGATGAAAGG) and Tt9G-R (5′→3′, GCGGCGGTCGACAAGACATTGGG) were used to amplify the coding sequence of *Tt*LPMO9G from the Twist synthesis vector, and the PCR product was purified with the GeneJET PCR purification kit (Thermo Scientific, Waltham, MA, USA). The remaining *Tt*LPMO9 genes were digested from the commercial Twist synthesis vectors; SalI and BstBI restriction enzymes were used for classic restriction digestion cloning into pPICZα A (Invitrogen, Life Technologies Corporation AS, Carlsbad, CA, USA). pPICZα A plasmids containing the genes fused to a C-terminal His_6_ tag, originally present in the pPICZα A vector, were subsequently transformed into E. coli DH5α for amplification. Restriction enzyme digestions and DNA sequencing (Eurofins, Hamburg, Germany) were used to verify that the various E. coli transformants carried the correct plasmids with the correct genes.

### Gene cloning of *Tt*LPMO9E.

The coding sequence of *Tt*LPMO9E (UniProt ID G2RGE5), including the native signal peptide, was codon optimized for expression in P. pastoris, inserted between an EcoRI cleavage site and a Kozak sequence at the 5′ end (GAATTCGAAAGC) and a stop codon and an Acc65I cleavage site (TAGGGTACC) at the 3′ end and synthesized by GenScript (Piscataway, NJ, USA). The gene was cloned into the pPink-GAP plasmid using restriction digestion, the resulting plasmid was then cloned into PichiaPink strain 4 (Invitrogen, Life Technologies Corporation AS) by following the manufacturer’s instructions, and the transformants were screened to select the strain with the highest protein production level by following the protocol described earlier (85).

### Heterologous expression and purification of *Tt*LPMO9A, -9B, -9G, -9T, and -9U.

Correct pPICZα A plasmids carrying the *lpmo* genes were transformed into P. pastoris SMD1168H (Invitrogen Life Technologies Corporation AS) according to the procedure reported by the iGEM Stockholm 2018 team (https://2018.igem.org/Team:Stockholm), with the following adaptions. In detail, SacI was first used to linearize the plasmids encoding *Tt*LPMO9A, *Tt*LPMO9B, *Tt*LPMO9G, and *Tt*LPMO9U, and BstXI was used for linearizing the *Tt*LPMO9T-encoding plasmid. The DNA was purified with the GeneJet PCR kit prior to yeast transformation. For preparing electrocompetent P. pastoris cells, an overnight culture was grown in 5 mL yeast extract–peptone–dextrose medium (YPD) to an optical density at 600 nm (OD_600_) of approximately 4.0 at 30°C and 200 rpm. Of this preculture, 25 μL was reinoculated into 200 mL YPD in a 1-liter baffled shake flask, followed by incubation under the same conditions for 23 h until the OD_600_ was approximately 1.5. The cells were then centrifuged at 900 × *g* and 4°C for 5 min. The pellet was resuspended in 200 mL ice-cold sterile water, followed by another round of centrifugation and resuspension in 100 mL ice-cold sterile water. The cells were then centrifuged again and resuspended in 8 mL ice-cold sterile water, followed by another centrifugation and resuspension in 8 mL ice-cold 1 M sorbitol. After another round of centrifugation, the cells were resuspended in 800 μL ice-cold 1 M sorbitol. The cells were kept on ice as 80-μL aliquots and used for transformation on the same day. Linearized plasmids (1.5 to 5 μg) were transformed into P. pastoris SMD1168H using electroporation in 0.2-cm cuvettes, using pulsing parameters developed for S. cerevisiae. Ice-cold 1 M sorbitol (1 mL) was added to the cuvette directly after pulsing. The cuvette contents were then transformed to a sterile tube and incubated at 30°C without shaking for 3 h. Next, 100 or 200 μL of this mix was plated on YPD plates with sorbitol (YPDS) either with 100 μg/mL or 200 μg/mL Zeocin, followed by incubation at 30°C for up to 7 days.

Correct clones were verified by colony PCR. For this, first the genomic DNA was extracted according to Lõoke et al. (86), and 1 μL of the final supernatant was used as a template in a PCR with standard 5′AOX and 3′AOX primers. Protein production was assessed using 96-well deep plates to screen for strains producing at a high level. For expressing each of the *lpmo* genes, five clones were tested in buffered complex methanol medium (BMMY) for 5 days at 800 rpm and 30°C with feeding of 0.5% (vol/vol) methanol at time zero and then after every 24 h. Precultures were grown in buffered complex glycerol medium (BMGY) overnight, washed, and resuspended in BMMY to reach an OD_600_ of approximately 1. The final volume was 800 μL in each well both for precultures and production cultures. The multiwell plates were covered with Breathe Easy sealing membranes (Sigma-Aldrich, MO, USA) to allow for oxygen exchange. Supernatants from the 5-day cultivations were screened using stain-free SDS-PAGE.

For scaling up the production of the LPMOs, 5-liter baffled shake flasks were used with 1 liter BMMY, and cultures were incubated at 29°C and 250 rpm. Precultures were grown in BMGY medium overnight, washed, and resuspended in BMMY to reach a starting OD_600_ between 0.5 and 1. Methanol was fed at time zero and then every 24 h at 1% (vol/vol) for induction of protein production. Sorbitol (1%, wt/vol) was added at time zero and every 24 h afterwards to the flask with the *Tt*LPMO9U-producing strain and at time points 72 h and 90 h to the *Tt*LPMO9A-, *Tt*LPMO9B-, *Tt*LPMO9G-, and *Tt*LPMO9T-producing strain flasks. This was done as it was first shown to aid the production of *Tt*LPMO9U, as described in Results. The cells were cultivated for up to 5 days (except for *Tt*LPMO9U production, which was ended after 72 h, as described above). The supernatants were harvested by centrifuging and filtering using Nalgene Rapid-Flow sterile bottle-top filters (Thermo Scientific). Concentration of the supernatants prior to protein purification was done by cross-filtration with a Minimate tangential flow filtration capsule (Pall Corporation, NY, USA), having a polyether sulfone membrane with a 10-kDa cutoff.

The enzymes were purified using gravity flow-immobilized metal chelate affinity chromatography. Protein purity was confirmed (Fig. S4a) by Mini-PROTEAN SDS-PAGE stain-free precast gels (Bio-Rad, CA, USA). The ladder was Precision Plus protein unstained standard. After elution from the column, LPMO-containing fractions were pooled, followed by buffer exchange to 50 mM BisTris-HCl buffer, pH 6.5, and concentration using either 3-kDa Amicon Ultra (Sigma-Aldrich, MO, USA) or 10-kDa Pierce (Sigma-Aldrich, MO, USA) protein concentration centrifugal tubes. Protein concentrations were determined by measuring absorbance at 280 nm, using the following extinction coefficients, calculated using Expasy’s ProtParam tool: *Tt*LPMO9G, 53,455 M^−1^·cm^−1^; *Tt*LPMO9E, 58,120 M^−1^·cm^−1^; *Tt*LPMO9U, 66,015 M^−1^·cm^−1^; *Tt*LPMO9T, 45,505 M^−1^·cm^−1^; *Tt*LPMO9A, 37,150 M^−1^·cm^−1^; and *Tt*LPMO9B, 43,860 M^−1^·cm^−1^. The enzyme solutions were filtered through 0.22-μm Millex-GV filters (Merck Millipore, MA, USA) and kept at 4°C for short-term and at −20°C for long-term storage.

### Deglycosylation.

*Tt*LPMO9B was deglycosylated under nondenaturing conditions by incubating 47 μg of purified LPMO with 2 μL of PNGase F (New England Biolabs, MA, USA) in Glycobuffer 2 (50 mM Na-phosphate, pH 7.5) for 30 min at 25°C and then for 16 h at 37°C, 800 rpm. The final reaction volume was 20 μL. *Tt*LPMO9U was also deglycosylated under nondenaturing conditions by incubating 12 μg of the purified LPMO with 1 μL of deglycosylation mix II (New England Biolabs, MA, USA) in deglycosylation mix buffer 1 (50 mM Na-phosphate, pH 7.5) for 16 h at 37°C, 800 rpm. The final reaction volume was 10 μL.

### Heterologous expression and purification of *Tt*LPMO9E.

As no methanol induction was needed to produce *Tt*LPMO9E, the preculture and production culture were both done in YPD medium. The strain was grown for production for 4 days. Other conditions and parameters were the same as those for the strains mentioned above. The enzyme was purified by ion-exchange chromatography, using an Äkta Purifier system equipped with a HiTrap Q FF 5 mL anion exchange column (both from Cytiva Life Sciences, MA, USA). The start buffer was 20 mM Tris-HCl, pH 8.0, and the elution buffer was 20 mM Tris-HCl, pH 8.0, containing 1 M NaCl. The purity of the enzyme was confirmed by stain-free SDS-PAGE, like for other *Tt*LPMO9s. Concentrating the enzyme, measuring its concentration, and filtration and storage were done as described above for the other five LPMOs.

### Detection of H_2_O_2_ production.

Production of H_2_O_2_ by the LPMOs in the presence of a reducing agent and molecular oxygen was measured using the Amplex Red assay adapted from Kittl et al. (65). The reaction mixes (100 μl) contained 3 μM LPMO, 50 μM ascorbic acid (AscA), 0.5 U horseradish peroxidase, 100 μM Amplex Red in 50 mM BisTris-HCl buffer, pH 6.5. The LPMOs were preincubated with a 0.5 molar equivalent of CuSO_4_ for a minimum of 30 min before initiating the reactions by the addition of AscA. The absorbance at 563 nm was monitored over time using a Varioscan LUX plate reader (Thermo Fisher Scientific) at 30°C for 4,000 s, and the absorbance was measured every 22 s.

### Detection of H_2_O_2_ consumption in reactions with 2,6-dimethoxyphenol.

To test peroxidase-like activity of the LPMOs, we used an assay adapted from Breslmayr et al. (66). The reaction mixtures (100 μL) contained 3 μM LPMO, 50 μM 2,6-DMP, 100 μM H_2_O_2_ in 50 mM BisTris-HCl buffer, pH 6.5. The LPMOs were preincubated with a 0.5 molar equivalent of CuSO_4_ for a minimum of 30 min before initiating the reactions by adding the LPMO to the buffer solution containing 2,6-DMP and H_2_O_2_. The absorbance at 469 nm was monitored using a Varioscan LUX plate reader (Thermo Fisher Scientific) at 30°C for 3,600 s. The absorbance in each well was measured every 30 s.

### LPMO reactions with polysaccharide substrates.

All LPMO reactions with polysaccharide substrates were carried out for 16 h at 1,000 rpm and 40°C in 1.5-mL Eppendorf tubes with a 100-μL final reaction volume, except for reactions with sulfite-pulped spruce, where the final volume was 500 μL. All reaction mixtures contained 1 μM LPMO in 50 mM BisTris-HCl buffer, pH 6.5, and were initiated by the addition of 1 mM GA. The substrate concentrations were 0.4% (wt/vol) for PASC, 0.2% (wt/vol) for Avicel, and 1% (wt/vol) for sulfite-pulped spruce. For reactions with mixed substrates, both the PASC and the hemicellulose concentrations were 0.2% (wt/vol). PASC was prepared from Avicel as described previously (87). Sulfite-pulped spruce was from Borregaard AS (Borregaard, Norway); Avicel PH-101 was from Sigma-Aldrich; beechwood xylan was from Apollo Scientific (Cheshire, UK). Konjac glucomannan (KGM), cellopentaose (Glc_5_), low-viscosity wheat flour arabinoxylan (WAX), and tamarind xyloglucan (TXG) were from Megazyme (Bray, Ireland). Spruce arabinoglucuronoxylan (AGX) and acetylated galactoglucomannan (acGGM) as well as acetylated glucuronoxylan (acGX) from birch were produced in-house as described previously (76). Reactions were stopped by boiling for 10 min followed by filtering using 0.45-μm filter plates (Millipore, Darmstadt, Germany) prior to analytics.

### Product analysis by HPAEC-PAD.

LPMO products were analyzed by high-performance anion-exchange chromatography with pulsed amperometric detection (HPAEC-PAD). A Dionex ICS5000 system equipped with a CarboPac PA200 analytical column (3 by 250 mm) and a CarboPac PA200 guard column (3 by 50 mm) (Dionex, Sunnyvale, CA, USA) was used. Prior to injection, the samples were diluted two times in Milli-Q water. The injection volume was 5 μL. A multistep 39-min gradient with increasing amounts of eluent B was used to elute the products. The eluents were the following: A, 0.1 M NaOH; B, 0.1 M NaOH with 1 M Na-acetate. The gradient was linear between 0 and 5.5% B over 4.5 min (curve 5), linear between 5.5 and 15% B over 9 min (curve 5), convex upward between 15 and 100% B over 16.5 min (curve 4), concave upward between 100 and 0% B over 0.05 min (curve 8), and maintained at 0% B (reconditioning) for 9 min (curve 5). The oxidized cello-oligosaccharide standards were prepared by treating 0.05 g/liter Glc_2_-Glc_5_ with 1 μM cellobiose dehydrogenase from *Myriococcum thermophilum* (*Mt*CDH; GenBank accession no. EF492052.3) (88, 89) in 50 mM Na-acetate buffer, pH 5.0, at 40°C for 20 h.

### Product analysis by MALDI-TOF MS.

Matrix-assisted laser desorption/ionization time-of-flight mass spectrometry (MALDI-TOF MS) was conducted using an Ultraflex (Bruker Daltonics, Billerica, MA, USA) instrument equipped with a 337-nm laser in positive reflector mode, as described previously (12). After diluting samples four times in 5 mM BisTris-HCl with 15 mM NaCl (pH 6.0), 1.5 μL of sample was mixed with 1.5 μL matrix solution (10 mg/mL 2,5-dihydroxybenzoic acid in 30% [vol/vol] acetonitrile and 0.1% [vol/vol] trifluoroacetic acid) on an MTP 384 ground steel target plate (Bruker Daltonics) and dried with a blow-dryer. The laser intensity was 85%. Data were collected using the flexControl software, and data were analyzed using mMass (90).

### Data availability.

All relevant data are included in the main body or the supplemental material of this article.
