# Supplementary material for: Comparison of Six Lytic Polysaccharide Monooxygenases from Thermothielavioides terrestris Shows That Functional Variation Underlies the Multiplicity of LPMO Genes in Filamentous Fungi
Source: Appl Environ Microbiol. 2022 Mar 22;88(6):e00096-22. doi: 10.1128/aem.00096-22 (PMC8939357; doi:10.1128/aem.00096-22)
Supplement: Supplemental file 1 — Fig. S1 to S10. Download aem.00096-22-s0001.pdf, PDF file, 2.3 MB [file aem.00096-22-s0001.pdf]

*TtL*PMO9A:

MKLTTSVALLAAAGAQAHYTFPQTDINGQLSGEWVTIRETTNHYSHGPVTDVTSDQIRCYELNPGTPA
PQIATVQAGGTVTFTVDPSIQHPGPLQFYMAKAPSGQTAATFQGTGNVWFKIYEDGPSGLGTSNITWP
SSGKTEVSVKIPSCIAPGDYLLRVEHIALHSASTVGGAQFYLACAQLTVTGGTGTLNTGELVAFPGAY
SATDPGILFQLYWPIPTSYTNPGPAPVSC

*Tt*LPMO9B:

MKSFTIAALAALWAQEAAAHATFQDLWIDGVDYGSQCVRLPASNSPVTNVASDDIRCNVGTSRPTVKC
PVKAGSTVTIEMHQQPGDRSCANEAIGGDHYGPVMVYMSKVDDAVTADGSSGWFKVFQDSWAKNPSGS
TGDDDYWGTKDLNSCCGKMNVKIPEDIEPGDYLLRAEVIALHVAASSGGAQFYMSCYQLTVTGSGSAT
PSTVKFPGAYSASDPGILVNIHAPMSTYVVPGPTVYAGGSTKSAGSSCSGCEATCTVGSGPSATLTQP
TSTATSAPGGGGSGCTAAKYQQCGGTGYTGCTTCASGSTCSAVSPPYYSQCL

*TtL*PMO9E:

MLANGAIVFLAAALGVSGHYTWPRVNDGADWQQVRKADNWQDNGYVGDVTSPQIRCFQATPSPAPSVL
NTTAGSTVTYWANPDVYHPGPVQFYMARVPDGEDINSWNGDGAVWFKVYEDHPTFGAQLTWPSTGKSS
FAVPIPPCIKSGYYLLRAEQIGLHVAQSVGGAQFYISCAQLSVTGGGSTEPPNKVAFPGAYSATDPGI
LINIYYPVPTSYQNPGPAVFSC

*Tt*LPMO9G:

MKGLFSAAALSLAVGQASAHYIFQQLSINGNQFPVYQYIRKNTNYNSPVTDLTSDDLRCNVGAQGAGT
DTVTVKAGDQFTFTLDTPVYHQGPISIYMSKAPGAASDYDGSGGWFKIKDWGPTFNADGTATWDMAGS
YTYNIPTCIPDGDYLLRIQSLAIHNPWPAGIPQFYISCAQITVTGGGNGNPGPTALIPGAFKDTDPGY
TVNIYTNFHNYTVPGPEVFSCNGGGSNPPPPVSSSTPATTTLVTSTRTTSSTSSASTPASTGGCTVAK
WGQCGGNGYTGCTTCAAGSTCSKQNDYYSQCL

*Tt*LPMO9T:

MQLLVGLLVAAVAARAHYTFPRLVVNGQPEDKDWSVTRMTKNAQSKQGVQDPTSPDIRCYTSQTAPNV
ATVPAGATVHYISTQQINHPGPTQYYLAKVPEGSSAKTWDGSGAVWFKISTTMPYLDSNKQLVWPNQN
TYTTVNTTIPADTPSGEYLLRVEQIALHLASQPNGAQFYLACSQIQITGGGNGTPGPLVALPGAYKSN
DPGILVNIYSMQPGDYKPPGPPVWSG

*Tt*LPMO9U:

MKLHLAAFLGVITTPGAFAHQIHGILLVNGTETPEWKYVRDVAWEGAYKPEKYPNTEFFKTPPQTDIN
NPNITCGRNAFDSANKTETADILAGSEVGFRVSWDGNGKYGVFWHPGPGQIYLSRAPNDDLENYRGDG
DWFKIAIGAAVSNTEWLLWNKHDFNFTIPKTTPPGKYLMRIEQFMPSTVEYSQWYVNCAHVNIIGPGG
GTPTGFARFPGTYTVDDPGIKVPLNLIVNSGELPQDQLRLLEYKPPGPALWTG

Figure S1. Protein sequences of the six *Thermothielavioides terrestris* AA9 LPMOs used in this study. The signal peptide is marked in black, the AA9 LPMO domain in red, linker regions in grey and CBM1 domains in yellow. Note that the cloned *Tt*LPMO9A, 9B, 9G, 9T, and 9U genes also included a C-terminal His_6-tag which is not shown in this figure.

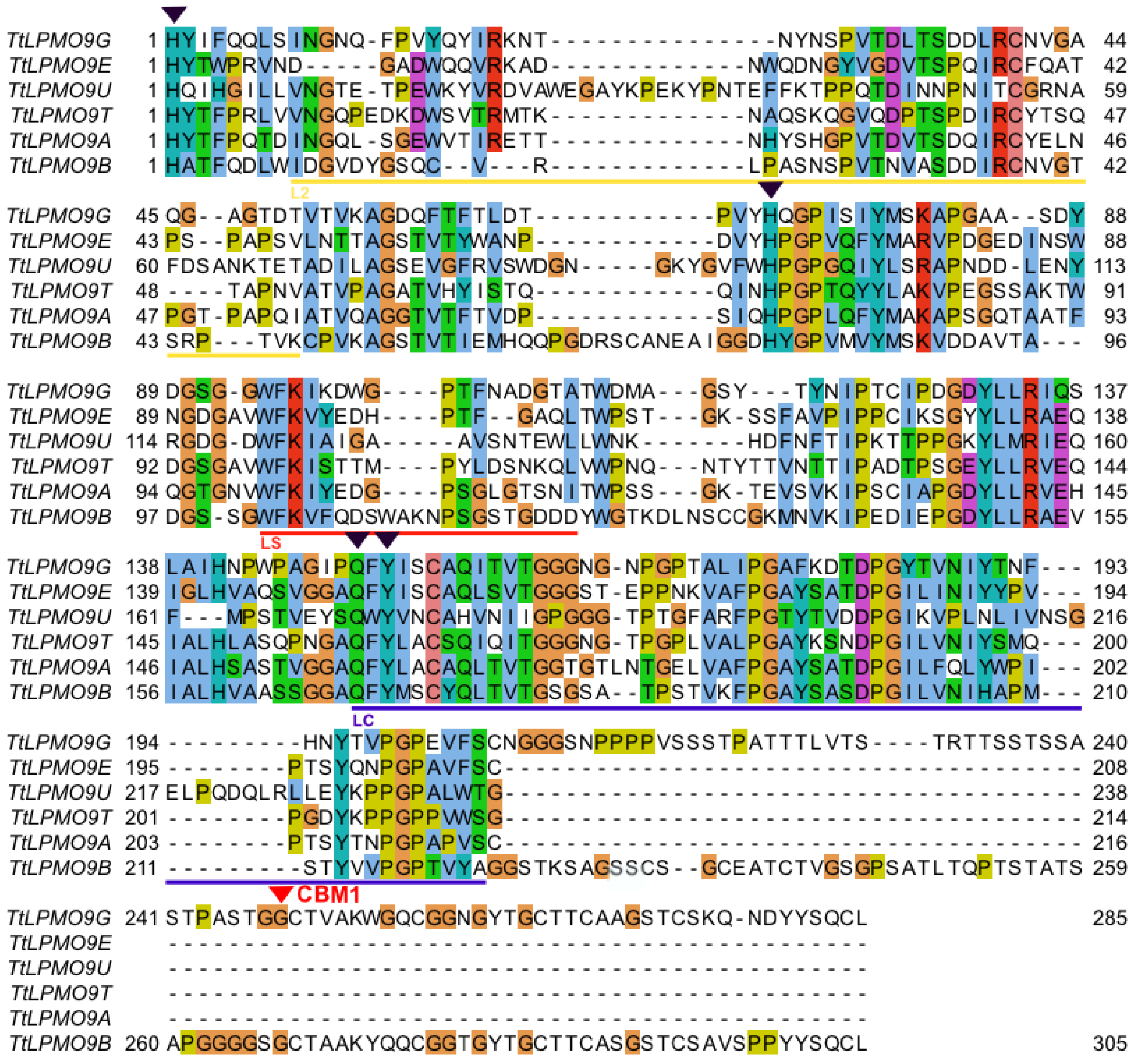

Figure S2. Multiple sequence alignment of the six *Thermothielavioides terrestris* AA9 LPMOs used in this study. The alignment was constructed with the Clustal Omega online tool with default parameters. The colors in the alignment indicate ClustalW color scheme. The loops L2, LS, and LC are marked according to *Tt*LPMO9E alignment in Borisova et al. (1). The two conserved histidine residues and a conserved tyrosine and glutamine in the primary and secondary copper coordination spheres are marked with black arrows. The start of the CBM1 domain in *Tt*LPMO9G and *Tt*LPMO9B is marked with the red arrow, based on the CBM1 domain of *Tr*Cel7B from *Trichoderma reesei* (2), and was detected with both Pfam and the Conserved Domain Database.

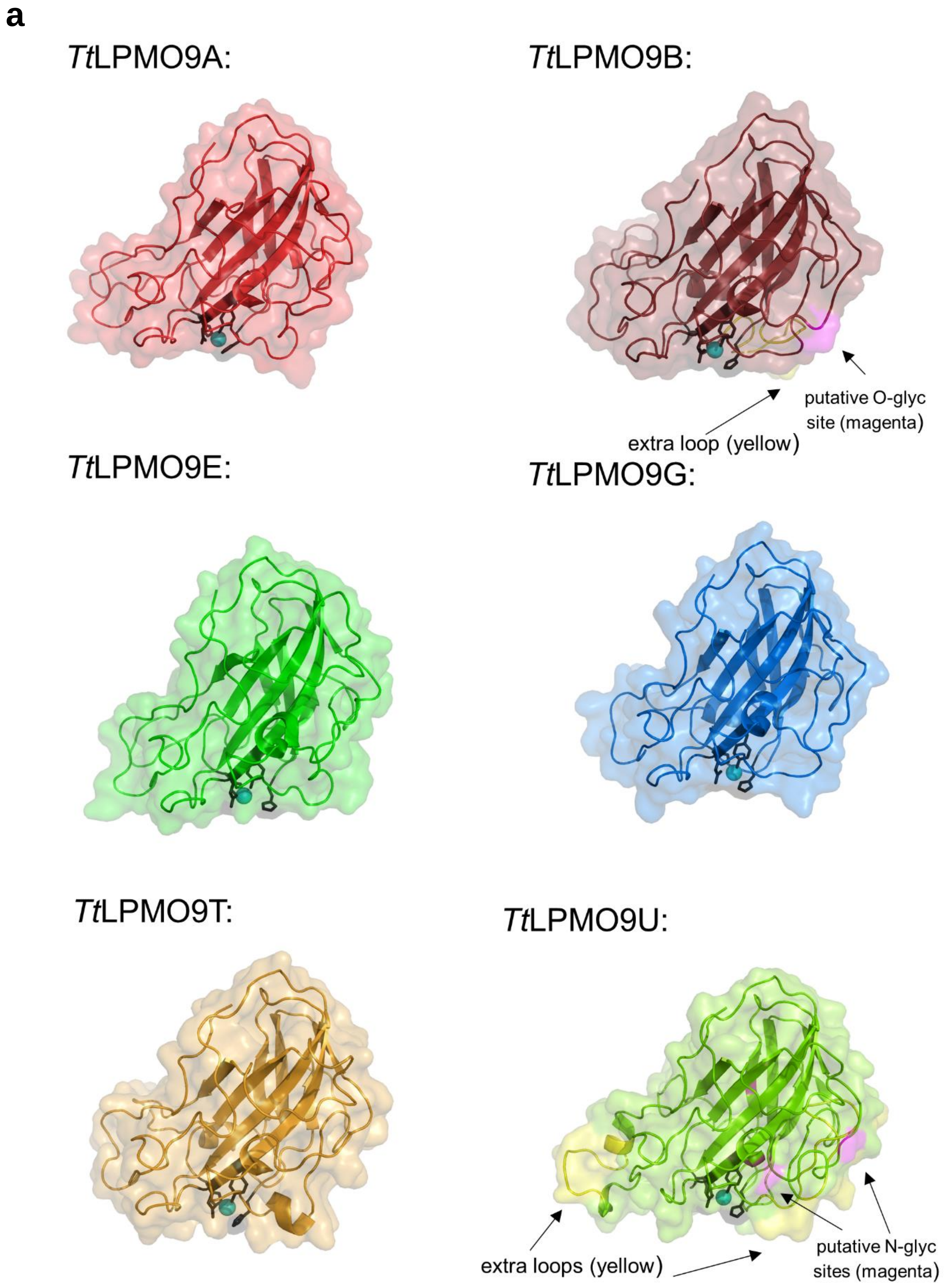

Figure S3. (Legend is given on the next page.)

b

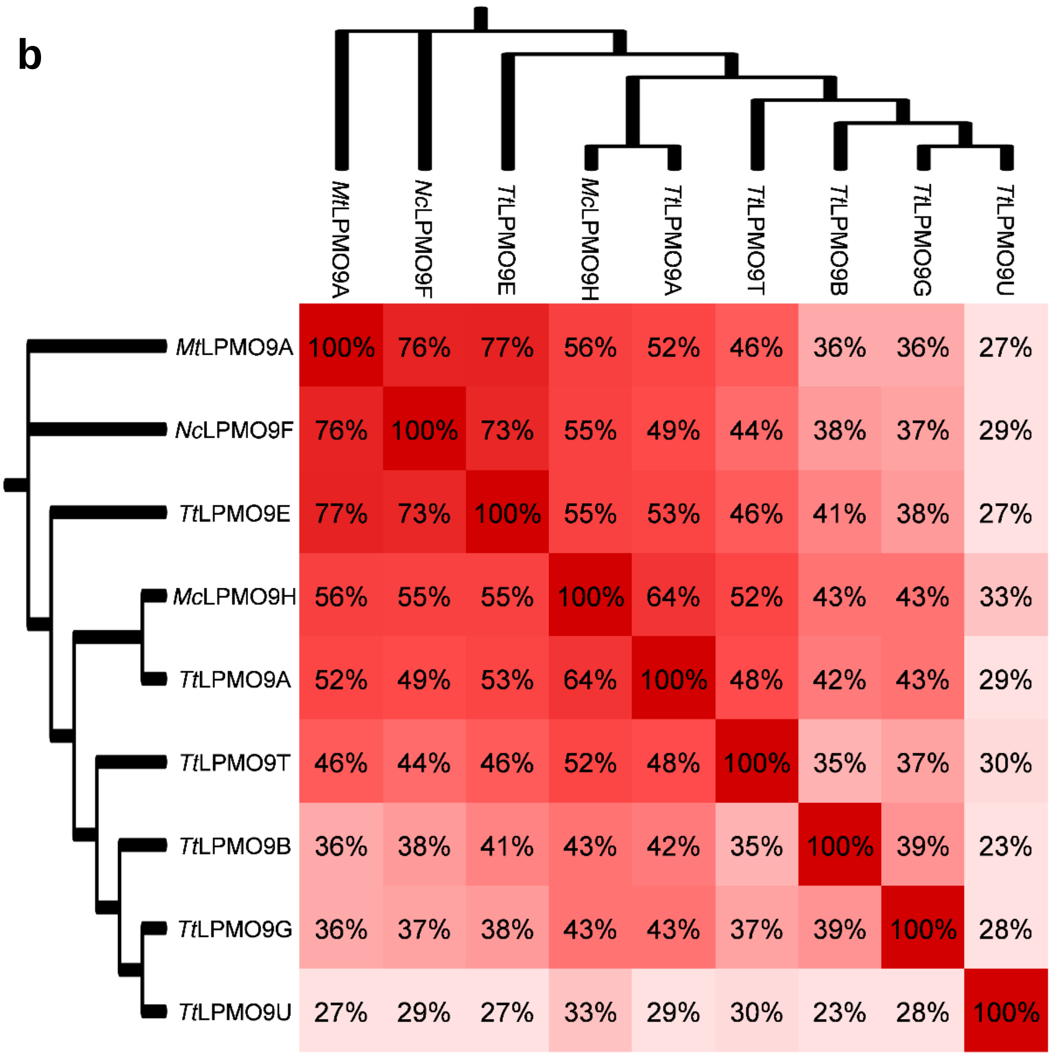

Figure S3. Structure models of the AA9 domains of five *Tt*LPMO9s and crystal structure of *Tt*LPMO9E (a) and phylogenetic relationships and sequence identities of selected AA9 LPMOs (b). The homology models were built using Phyre2 (3) for all except *Tt*LPMO9E, for which the solved crystal structure was obtained from PDB. Visualization was done using PyMoL (The PyMOL Molecular Graphics System, Version 0.99 Schrödinger, LLC). The Cu atom (in cyan in all structures) in the catalytic site was added to the structures by aligning it with the solved *Pc*LPMO9D crystal structure (PDB ID, 4B5Q). The two conserved histidines, and a tyrosine and a glutamine in the catalytic center are marked with black sticks in all structures. The extra two loops detected by multiple sequence alignment in *Tt*LPMO9U and one loop for *Tt*LPMO9B are marked in yellow. The putative O- and N-glycosylation sites of the AA9 domains that are possibly glycosylated and which might influence substrate binding are

marked in magenta. Note that *Tt*LPMO9G and 9B also carry putative glycosylation sites in their linker regions, which are not included in this figure. The glycosylation sites were predicted with the NetOGlyc 4.0 (4) and NetNGlyc 1.0 (5) tools. The matrix table shows the sequence identities of the catalytic domains of the six *Tt*LPMOs tested in this study and of the previously studied xylan-active AA9 LPMOs *Mt*LPMO9A, *Mc*LPMO9H and *Nc*LPMO9F. The sequence identities apply to the AA9 catalytic domain only, without signal peptides, linkers and CBMs. The phylogenetic consensus tree was built using ProtTest 3.4 using an Expresso (T-Coffee) MSA of the catalytic domains only.

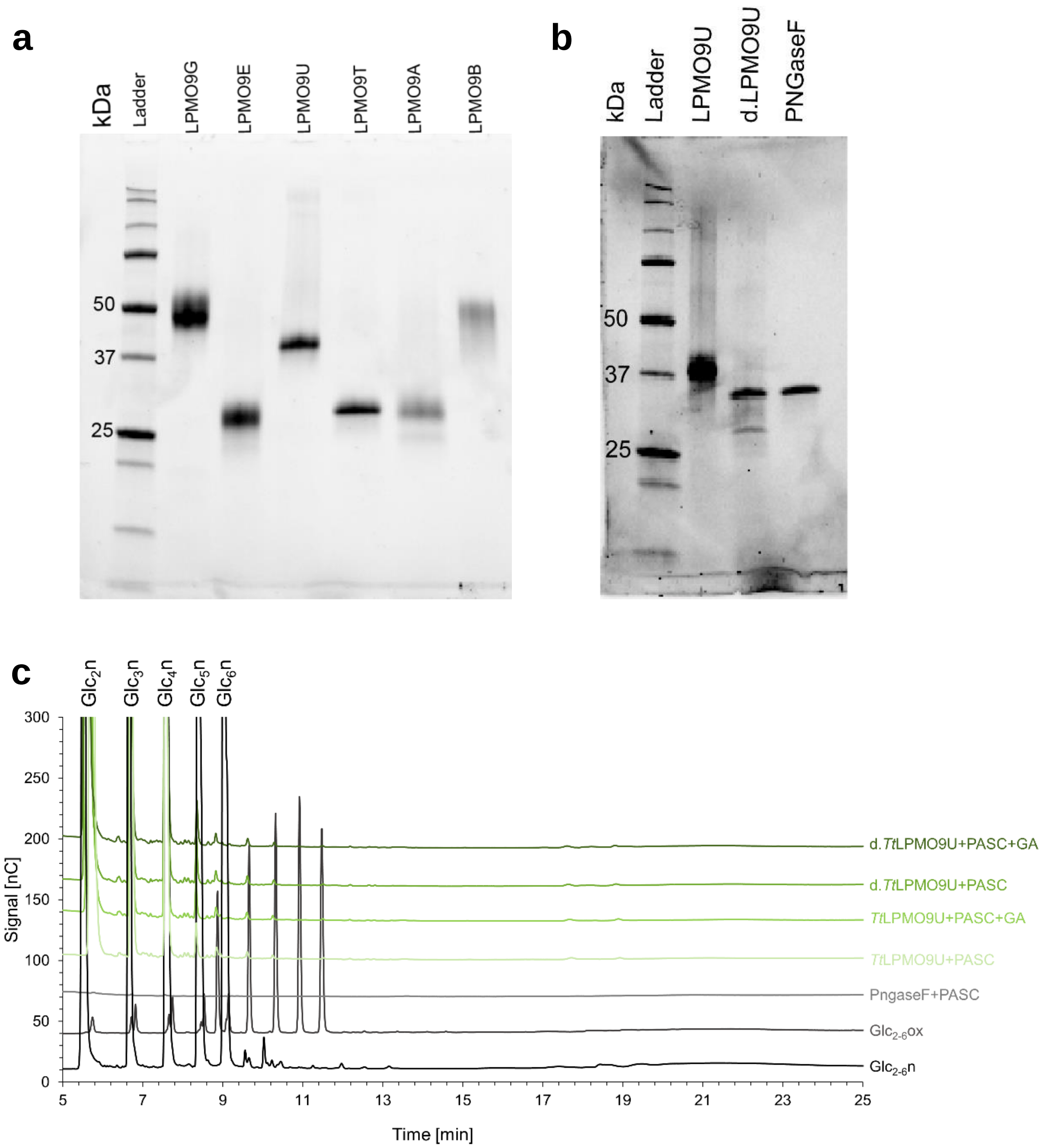

Figure S4. SDS-PAGE analysis of the purified *Tt*AA9 LPMOs (a) and the impact of deglycosylation on *Tt*LPMO9U (b, c). The LPMOs were purified using gravity flow IMAC and analyzed by SDS-PAGE using a stain-free gel as described in the Materials and methods section. Note that the double protein bands at 24 and 26 kDa for *Tt*LPMO9A have appeared for this enzyme in several rounds of production and always after the purification step as well, indicating, for instance, possible differential glycosylation. Panel b shows the impact of treating *Tt*LPMO9U with PNGase F and indicates that de-N-glycosylation was achieved. Panel c

shows product formation by 1mM of non-treated or de-glycosylated LPMO (d.LPMO9U) in standard overnight reactions with PASC and indicates that both enzyme forms are inactive.

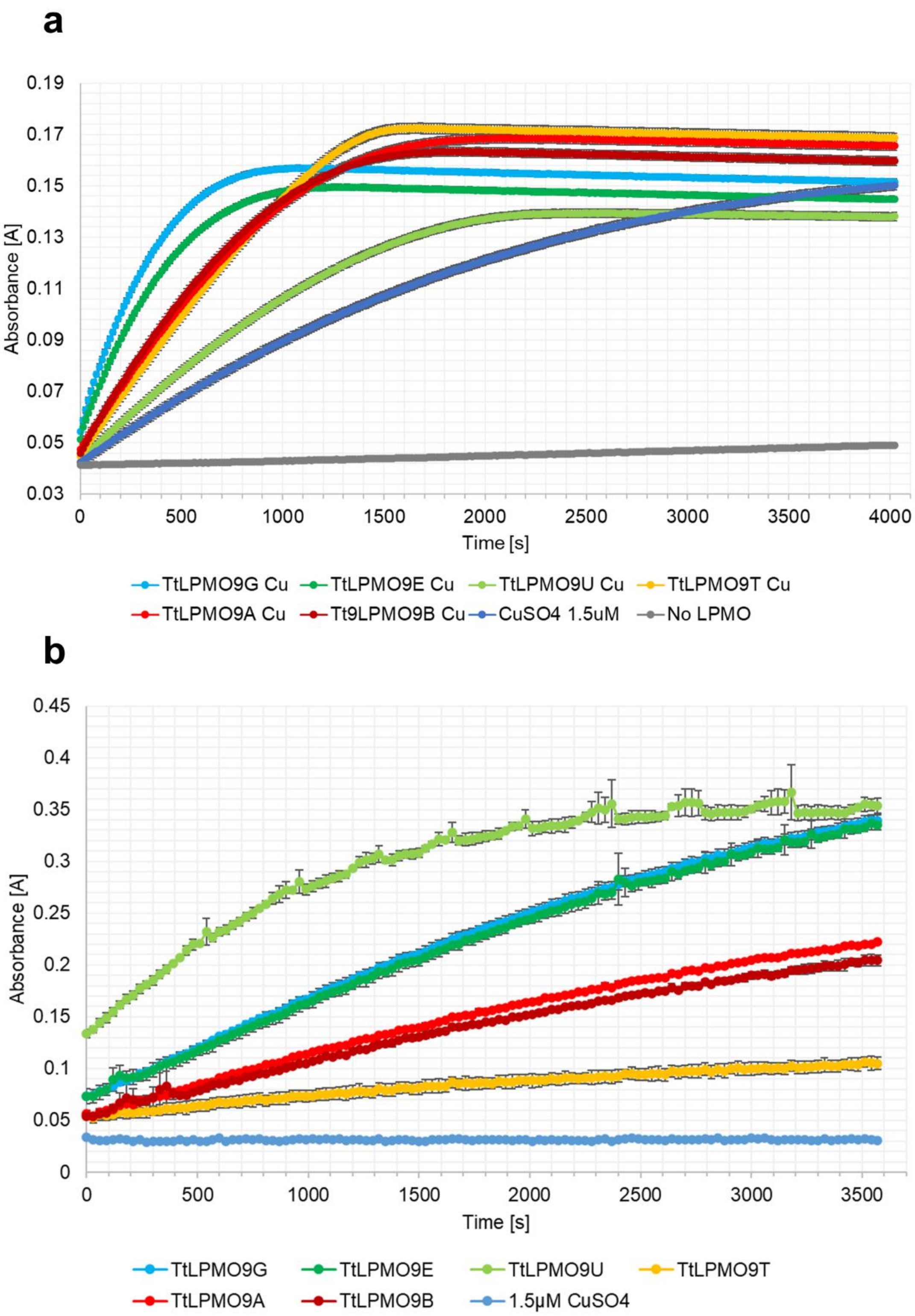

Figure S5. H_2O_2 accumulation (a) and peroxygenase-like H_2O_2 consumption (b) by the *Tt*LPMO9s. The reactions in **panel a** consisted of the following: 3 µM of LPMO, 50 µM ascorbic acid (added last to initiate catalysis), 0.5U horseradish peroxidase, 100 µM Amplex Red and

50 mM BisTris/HCl buffer at pH 6.5. The LPMOs were incubated with 0.5 equimolar amounts of $CuSO_4$ for a minimum of 30 min prior to the reaction. As a negative control, buffer was added instead of LPMO. In addition, a copper-control containing 1.5 µM $CuSO_4$ was used to demonstrate the differences between free copper and the LPMOs. In panel **b**, the reactions consisted of the following: 3 µM of LPMO, 1 mM 2,6-dimethoxyphenol, 100 µM H_2O_2 and 50 mM BisTris/HCl buffer at pH 6.5. The LPMOs were incubated with 0.5 equimolar amounts of $CuSO_4$ for minimum 30 min prior to the reaction. As a negative control, 1.5 µM $CuSO_4$ was used instead of LPMO. For both panels, error bars indicate standard deviation of three technical replicates.

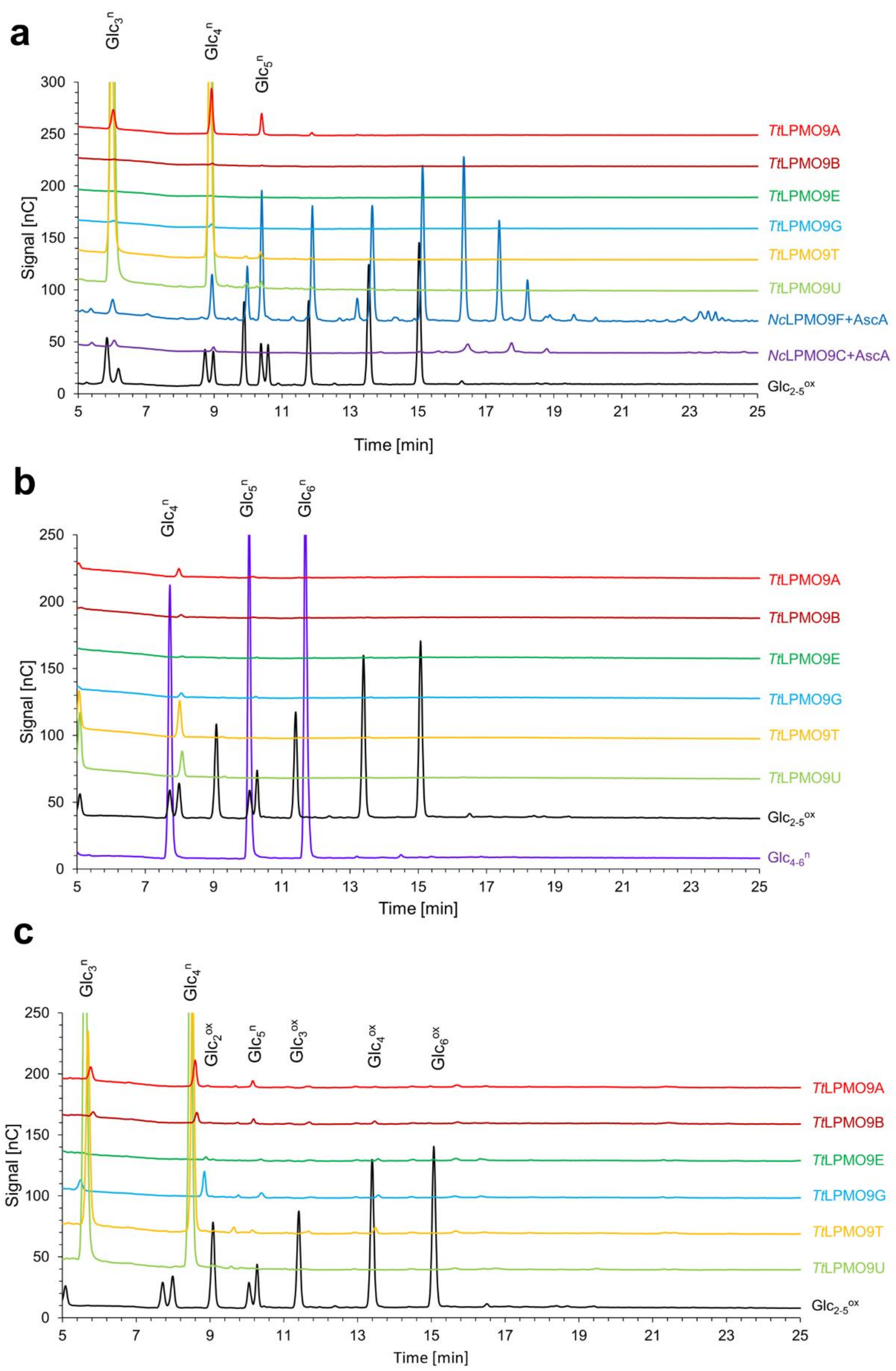

Figure S6. HPAEC-PAD chromatograms of control reactions without reductant for *Tt*LPMO9s on PASC (a), Avicel (b) and pulp fibres (c). LPMO (1 µM) was incubated with 0.4% (w/v) PASC (panel a), 0.2% (w/v) Avicel (panel b), and 1% (w/v) pulp fibres (panel

without any reductant, as a negative control, in 50 mM BisTris/HCl buffer at pH 6.5. *Tt*LPMO9s were incubated with 0.5 equimolar concentration $CuSO_4$ minimum 30 min prior to initiating the reaction by adding the LPMO to the buffered substrate. Reactions were carried out in 100 µL (PASC, Avicel) or 500 µL (pulp fibres) final volume for 16 h at 1000 rpm and 40°C.

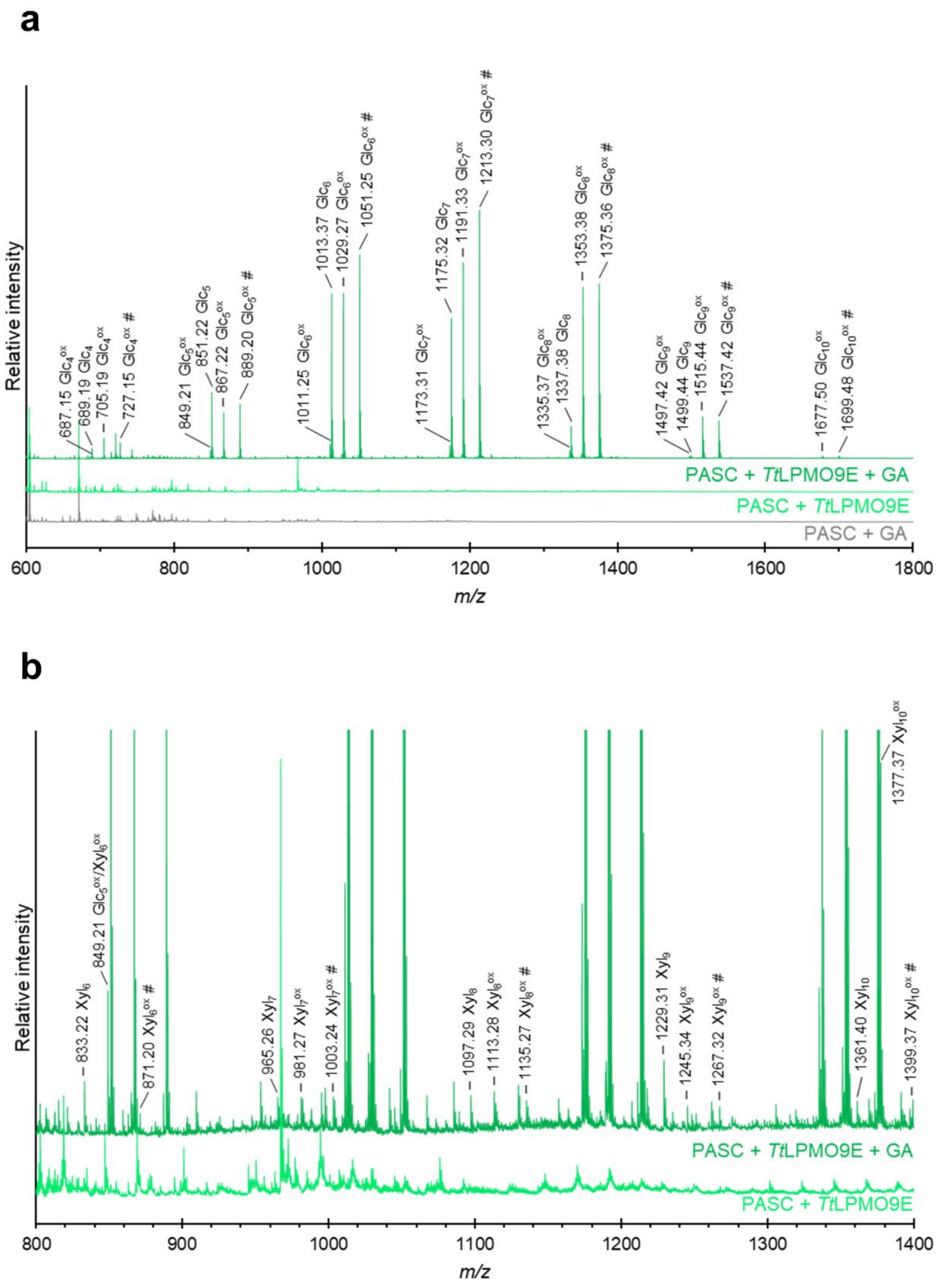

Figure S7. MALDI-TOF MS spectra from *Tt*LPMO9E reaction on PASC. Panel **a** shows the native and oxidized cello-oligosaccharides released by *Tt*LPMO9E in reactions with PASC. The reactions contained 1 µM LPMO and 0.4% (w/v) PASC and were incubated in the presence or absence of 1 mM gallic acid (GA), in 50 mM BisTris/HCl buffer pH 6.5 for 16 h at

40°C and 1000 rpm. Panel **b** shows a zoom in of the range *m/z* = 800-1400 of the same spectrum that shows the presence of small amounts of solubilized oxidized and native xylo-oligosaccharides produced by the LPMO. The Na^+-adducts of oligosaccharides are labelled; oxidized oligosaccharides are marked with “ox”, and the Na^+-salt of the (C1-)oxidized oligosaccharides are marked with #.

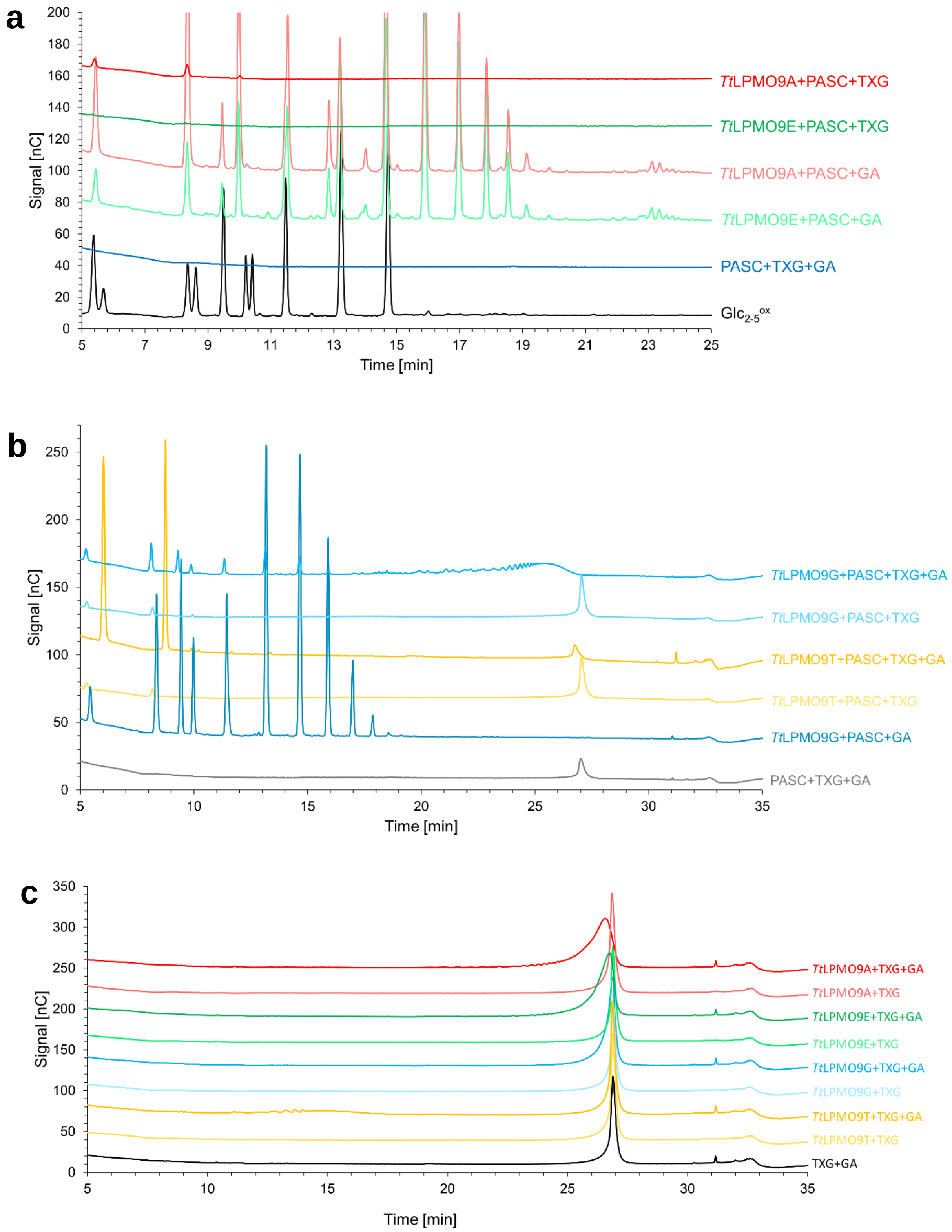

Figure S8. HPAEC-PAD chromatograms for (a) *Tt*LPMO9A and *Tt*LPMO9E acting on a TXG-PASC mixture in the absence of reductant, (b) *Tt*LPMO9G and *Tt*LPMO9T acting on a TXG-PASC mixture under various conditions, and (c) all LPMOs acting on TXG

alone or in the presence of reductant. LPMO (1µM) was incubated with 0.2% PASC (w/v) and 0.2% TXG (w/v) or with 0.4% (w/v) TXG (Fig. S8c), in 50mM BisTris/HCl buffer, pH 6.5. *Tt*LPMOs were incubated with 0.5 equimolar concentration $CuSO_4$ minimum 30 min prior to initiating the reaction by adding the LPMO to the buffered substrate. Reactions were carried out in 100µL final volume for 16h at 1000rpm and 40°C.

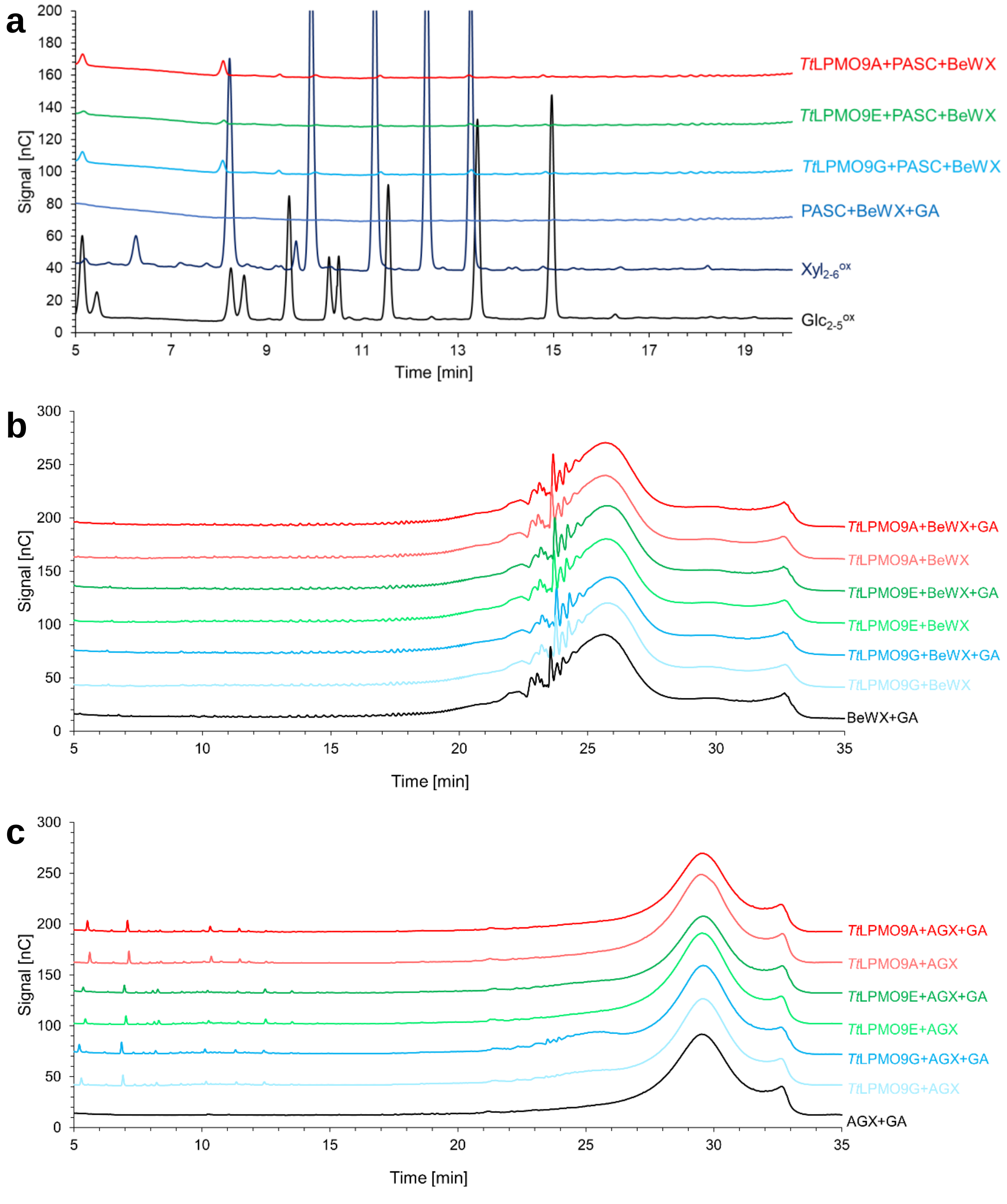

Figure S9. (Legend is given on the next page.)

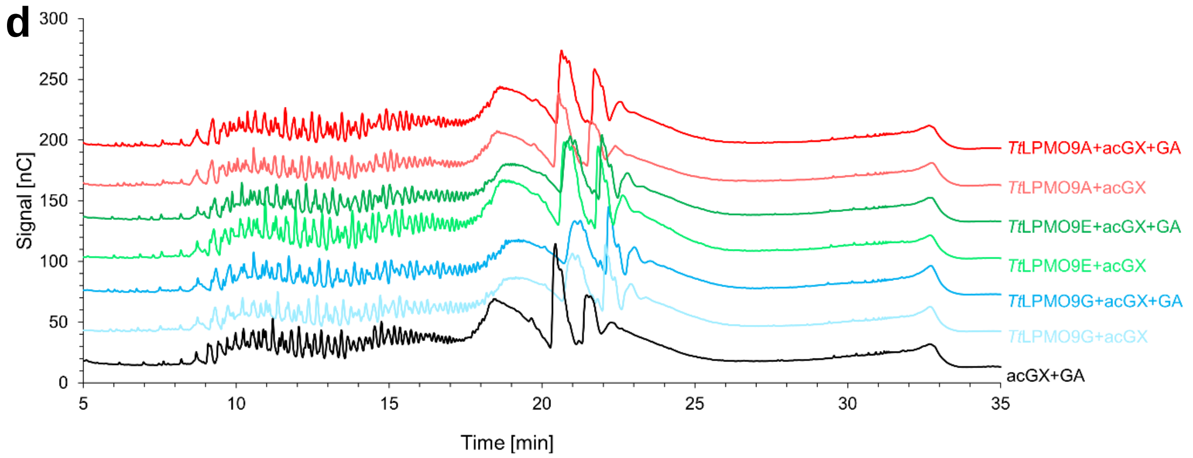

Figure S9. HPAEC-PAD chromatograms for control reactions with xylan-active *Tt*LPMO9s acting on a mixture of BeWX and PASC in the absence of reductant (a) and for reactions with BeWX (b), AGX (c) or acGX (d) only in the absence or presence of reductant. LPMO (1 µM) was incubated with 0.2% (w/v) PASC and 0.2% (w/v) BeWX or with 0.4% (w/v) xylan substrate (Fig S9b-d) in 50 mM BisTris/HCl, pH 6.5. The *Tt*LPMO9s were incubated with 0.5 equimolar concentration $CuSO_4$ for minimum 30 min prior to initiating the reaction by adding the LPMO to the buffered substrate. Reactions were carried out in 100 µL final volume for 16 h at 1000 rpm and 40 °C.

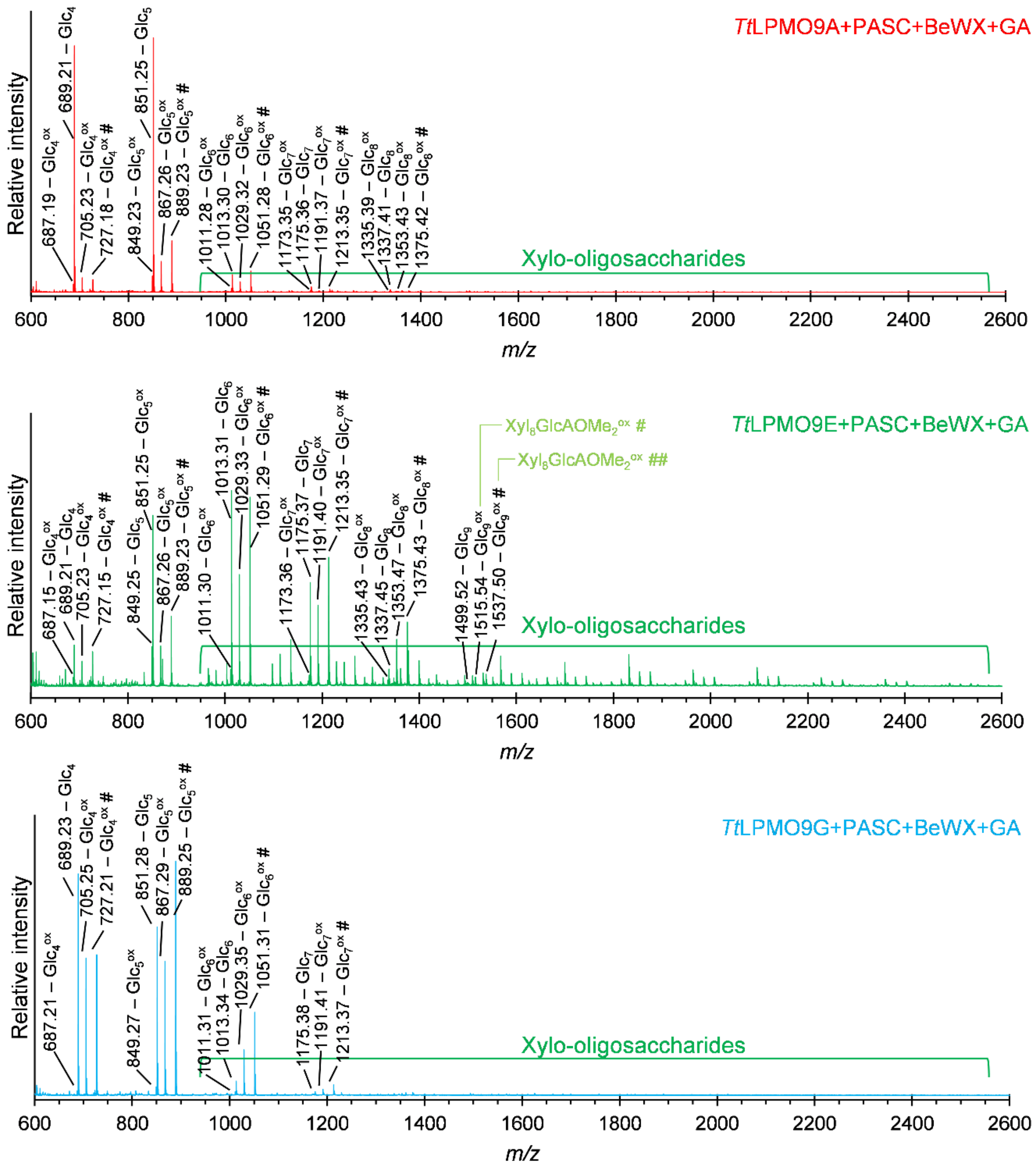

Figure S10. MALDI-TOF MS spectra of products generated by *Tt*LPMO9A, E and G in reaction mixtures containing both PASC and BeWX. All labelled peaks are sodium adducts. Sodium salts (+22 per sodium), which can be formed through binding to the carboxylic group at the C1-oxidized sugar Glc1A or Xyl1A and at the Glc*p*AOMe substitutions are annotated with # or ## for one or two Na^+ ions respectively. Oxidized products (anhydrated, Δ*m/z*=-2; hydrated, Δ*m/z*=+16) are labelled “ox”. The *m/z* range for which oxidized glucuronoxylan-oligomers were detected is marked with a green bracket.

References

1. Borisova AS, Isaksen T, Dimarogona M, Kognole AA, Mathiesen G, Várnai A, Røhr ÅK, Payne CM, Sørlie M, Sandgren M, Eijsink VGH. Structural and functional characterization of a lytic polysaccharide monooxygenase with broad substrate specificity. J Biol Chem. 2015;290(38):22955–69.

2. Mattinen M-L, Linder M, Drakenberg T, Annila A. Solution structure of the cellulose-binding domain of endoglucanase I from Trichoderma reesei and its interaction with cello-oligosaccharides. Eur J Biochem. 1998;256(2):279–86.

3. Kelley LA, Mezulis S, Yates CM, Wass MN, Sternberg MJE. The Phyre2 web portal for protein modeling, prediction and analysis. Nat Protoc. 2015;10(6):845–58.

4. Steentoft C, Vakhrushev SY, Joshi HJ, Kong Y, Vester-Christensen MB, Schjoldager KT-BG, Lavrsen K, Dabelsteen S, Pedersen NB, Marcos-Silva L, Gupta R, Bennett EP, Mandel U, Brunak S, Wandall HH, Levery SB, Clausen H. Precision mapping of the human O-GalNAc glycoproteome through SimpleCell technology. EMBO J. 2013;32(10):1478–88.

5. Gupta R, Brunak S. Prediction of glycosylation across the human proteome and the correlation to protein function. Pac Symp Biocomput. 2002;310–22.
